# Strong metal-support interaction induced by Pt-O-Bi bonding in mesoporous anatase TiO_2_ for base-free catalytic biomass valorization

**DOI:** 10.1093/nsr/nwaf327

**Published:** 2025-08-26

**Authors:** Haimei Xu, Chao Feng, Yunzhao Fan, Huawei Geng, Qisong Yi, Xiaoning Li, Yameng Fan, Yibo Ma, Baohua Jia, Yuanshuai Liu, Valentin Valtchev, Tianyi Ma

**Affiliations:** Qingdao Institute of Bioenergy and Bioprocess Technology, Chinese Academy of Sciences, Shandong Energy Institute, Qingdao Energy Shandong Laboratory, Qingdao 266101, China; Centre for Atomaterials and Nanomanufacturing (CAN), School of Science, RMIT University, Melbourne VIC 3000, Australia; Qingdao Institute of Bioenergy and Bioprocess Technology, Chinese Academy of Sciences, Shandong Energy Institute, Qingdao Energy Shandong Laboratory, Qingdao 266101, China; Qingdao Institute of Bioenergy and Bioprocess Technology, Chinese Academy of Sciences, Shandong Energy Institute, Qingdao Energy Shandong Laboratory, Qingdao 266101, China; Qingdao Institute of Bioenergy and Bioprocess Technology, Chinese Academy of Sciences, Shandong Energy Institute, Qingdao Energy Shandong Laboratory, Qingdao 266101, China; Qingdao Institute of Bioenergy and Bioprocess Technology, Chinese Academy of Sciences, Shandong Energy Institute, Qingdao Energy Shandong Laboratory, Qingdao 266101, China; University of Chinese Academy of Sciences, Beijing 100049, China; Centre for Atomaterials and Nanomanufacturing (CAN), School of Science, RMIT University, Melbourne VIC 3000, Australia; ARC Industrial Transformation Research Hub for Intelligent Energy Efficiency in Future Protected Cropping (E2Crop), Melbourne VIC 3000, Australia; Centre for Atomaterials and Nanomanufacturing (CAN), School of Science, RMIT University, Melbourne VIC 3000, Australia; ARC Industrial Transformation Research Hub for Intelligent Energy Efficiency in Future Protected Cropping (E2Crop), Melbourne VIC 3000, Australia; Centre for Atomaterials and Nanomanufacturing (CAN), School of Science, RMIT University, Melbourne VIC 3000, Australia; ARC Industrial Transformation Research Hub for Intelligent Energy Efficiency in Future Protected Cropping (E2Crop), Melbourne VIC 3000, Australia; Centre for Atomaterials and Nanomanufacturing (CAN), School of Science, RMIT University, Melbourne VIC 3000, Australia; ARC Industrial Transformation Research Hub for Intelligent Energy Efficiency in Future Protected Cropping (E2Crop), Melbourne VIC 3000, Australia; Qingdao Institute of Bioenergy and Bioprocess Technology, Chinese Academy of Sciences, Shandong Energy Institute, Qingdao Energy Shandong Laboratory, Qingdao 266101, China; Normandie université, ENSICAEN, UNICAEN, CNRS, Laboratoire Catalyse et Spectrochimie, Caen 14050, France; Centre for Atomaterials and Nanomanufacturing (CAN), School of Science, RMIT University, Melbourne VIC 3000, Australia; ARC Industrial Transformation Research Hub for Intelligent Energy Efficiency in Future Protected Cropping (E2Crop), Melbourne VIC 3000, Australia

**Keywords:** biomass valorization, base-free 5-hydroxymethylfurfural oxidation, single-atom, strong metal-support interaction, mesoporous anatase TiO_2_

## Abstract

The exceptional catalytic activity of bimetallic or trimetallic catalysts has been demonstrated in various reactions. However, in-depth understanding of the intricate metal-support interactions remains crucial yet challenging. Herein, a well-defined, highly active, Pt single-atom dominant Pt-Bi/TiO_2_ catalyst with mesoporous crystalline anatase structure was fabricated via an *in situ* one-pot evaporation-induced self-assembly method and employed for the base-free oxidation of biomass-derived 5-hydroxymethylfurfural (HMF) to value-added chemicals in aqueous media. Our findings demonstrate that Bi incorporation not only greatly reinforces the strong metal-support interaction between Pt and the support, but also benefits the Pt distribution, enabling the formation of single-atom Pt-O-Bi bonding, characterized by high-angle annular dark-field scanning transmission electron microscopy, X-ray photoelectron spectroscopy, carbon monoxide diffuse reflectance infrared Fourier transform spectroscopy and X-ray absorption spectroscopy analysis. Additionally, Bi incorporation results in remarkable increases in the catalyst's surface area and concentration of surface oxygen vacancies compared to the Pt/TiO_2_ counterpart. The changes in both geometric and electronic structures of the catalyst induced by Bi incorporation effectively reduce the dissociation energy of O_2_ into active oxygen species, the adsorption energy of HMF, as well as the activation barrier for its conversion to 2,5-diformylfuran, ultimately resulting in a significant enhancement in the conversion rate of HMF. These findings provide valuable insights into the rational design of efficient catalysts for biomass valorization.

## Teaser

This work develops a Pt single-atom dominant PtBi/TiO_2_ catalyst with high activity for base-free oxidation of biomass-derived HMF, and elucidates the complex metalsupport interactions involved.

## INTRODUCTION

The catalytic oxidation of 5-hydroxy methylf urf ural (HMF), a versatile platform compound derived from lignocellulosic biomass, has attracted significant attention for the production of various furan-containing fine chemicals, including 2,5-diformylfuran (DFF), 5-hydroxymethyl-2-furancarboxylic acid (HMFCA), 5-formyl-2-furancarboxylic acid (FFCA), and 2,5-furandicarboxylic acid (FDCA) [1–3]. In the aqueous-phase process, supported noble-metal-based catalysts are prevalently used in conjunction with alkaline solutions such as KOH(aq.), NaOH(aq.), Na_2_CO_3_(aq.), etc., to achieve appropriate activity and selectivity during the transformation of HMF [4–6]. The use of a basic medium, however, inevitably results in the formation of salts, thereby causing an extra increase in processing costs. Within this context, the so-called ‘base-free’ synthesis route using a solid-base-supported noble-metal catalyst, in an alkaline-free medium has been developed as an alternative, since it reduces chemical waste, simplifies the product recycling process, and minimizes the need for complex equipment and reagents.

Ag [7], Pd [8], Pt [9], Ru [10,11], and Au [12,13] are the commonly used noble-metal active sites in heterogenous ‘base-free’ oxidation of HMF to value-added products such as DFF [10,14], FFCA, HMFCA, and FDCA [15,16]. In particular, single-atom noble metal catalysts have attracted considerable attention due to their nearly 100% atom utilization, well-defined active sites, and high selectivity [17]. However, their practical application is often limited by low atom loading, uncontrollable atom distribution, and ambiguous interaction with the support, thereby hindering optimal catalytic performance [17]. To overcome these limitations, various strategies have been developed to synthesize single-atom catalysts with high density and locally ordered structure [17–19]. Nevertheless, when subjected to thermal or hydrothermal reaction conditions, these catalysts with single-phase noble metals supported on the surface often undergo deactivation caused by sintering of small metal particles into large aggregates, detachment of metal species from the support, deposition of coke or strong adsorbed intermediates blocking the accessibility of active sites, and occasionally poisoning. To address these challenges, significant efforts have been dedicated towards the development of dual-metal [20,21], multi-metal, and high-entropy alloy catalysts [22,23] which offer enhanced catalytic activity, selectivity, and stability. Emerging innovative fabrication techniques, such as laser scanning ablation [22], have enabled the preparation of uniform single-atom catalysts across the atomic-level design under ambient conditions. Among these systems, recent studies have revealed that bimetallic catalysts featuring dual-metal sites, such as Pt-Bi [6], Au-Cu [24], Ag-Pd [25], and Pd-Ni [26] have demonstrated superior activity when compared to their single-metal counterparts in the selective oxidation of HMF. This enhancement is primarily attributed to the synergistic effects between the two metals, which may alter the geometric and electronic structures of the catalyst.

In particular, Pt-Bi bimetallic catalysts have been proven effective in the oxidation of HMF to FDCA in base conditions [6]. A catalyst composed of single-atomic Pt anchored on Bi/C with a Bi to Pt molar ratio of 0.2 exhibited high catalytic activity with a FDCA yield of 83% when Na_2_CO_3_(aq.) was used as the base. Compared with Pt/C, Pt-Bi/C resulted in a 29% increase in FDCA yield. In addition, incorporating Bi has been found to effectively inhibit the aggregation of Pt nanoparticles while maintaining robust activity of the catalyst throughout the reaction [27]. Moreover, the presence of Bi in the Pt/C catalyst could also improve its resistance to oxygen poisoning, a common issue encountered in oxidation reactions [28]. Despite these advancements, the precise mechanism underlying the enhanced catalytic performance resulting from the combination of Bi and Pt remains unclear. Further investigation is imperative to uncover metal-support interactions and synergistic effects between Pt and Bi, elucidating how catalytic performance is influenced and facilitating further developments for improved or novel bimetallic oxidation catalysts.

Mesoporous materials, particularly those based on silica, carbon, or metal oxides, possess unique structural and chemical properties that make them an excellent support for bimetallic catalysts. Their large surface area, tunable pore size, chemical stability, and functional versatility provide abundant active sites, enhance mass transfer, and reduce coke formation [29–31], all of which contribute to improved selectivity and stability in biomass valorization processes such as HMF oxidation. However, to fully exploit these advantages, especially under harsh reaction conditions, high crystallinity is often essential. Crystalline mesoporous materials exhibit superior thermal and mechanical stability, which is critical for maintaining structural integrity and catalytic performance [32,33]. Despite their potential, synthesizing highly crystalline mesoporous metal oxides while preserving their meso-structure remains challenging.

In this study, an *in situ* one-pot evaporation-induced self-assembly (EISA) method was successfully employed to synthesize a Pt and Bi dual-metal incorporated catalyst (Pt-Bi/TiO_2_) with mesoporous crystalline anatase structure, featuring Pt single-atoms (SAs) predominantly incorporated in Bi/TiO_2_ support via Pt-O-Bi bonding. The as-prepared catalyst was subsequently evaluated for the ‘base-free’ oxidation of biomass-derived HMF and exhibited a remarkable enhancement in catalytic activity compared to the Pt/TiO_2_ and Bi/TiO_2_ catalysts prepared using the same method. To gain a deeper understanding of enhanced catalytic activity of Pt-Bi/TiO_2_, various characterization methods including powder X-ray diffraction (XRD), high-angle annular dark-field scanning transmission electron microscopy (HAADF-STEM), X-ray absorption spectroscopy (XAS), X-ray photoelectron spectroscopy (XPS), ultraviolet-visible (UV-vis) diffuse reflectance spectroscopy, electron paramagnetic resonance (EPR), and *in situ* diffuse reflectance infrared Fourier transform spectroscopy (DRIFT) using CO as the probe molecule, etc., have been employed. Through comprehensive characterizations, in conjunction with reaction kinetics and density functional theory (DFT) calculations, we provide valuable insights into the influence of Bi incorporation on the physicochemical properties of the catalyst, as well as the promotion effects of Bi on the ‘base-free’ catalytic oxidation of HMF.

## RESULTS AND DISCUSSION

### Textual structure analysis

**Figure 1.**
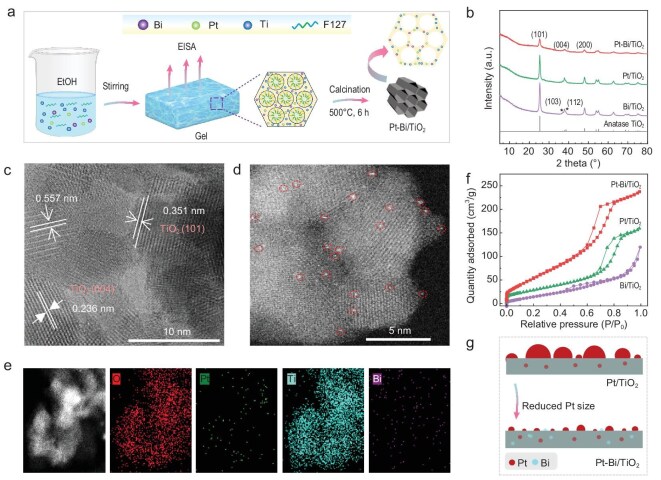
(a) Schematic illustration of the synthesis process of mesoporous TiO_2_-based materials via a facile one-pot evaporation-induced self-assembly (EISA) strategy; (b) XRD patterns of different TiO_2_-based samples; (c) HRTEM images of the Pt-Bi/TiO_2_; (d) HAADF-STEM image of Pt-Bi/TiO_2_; (e) HAADF-STEM image and elemental mapping images of Ti, Bi, and Pt on Pt-Bi/TiO_2_, respectively; (f) N_2_ adsorption/desorption isotherms of different TiO_2_-based samples; and (g) schematic diagram of Pt metal dispersion on Pt/TiO_2_ and Pt-Bi/TiO_2_.

As mentioned above, Pt-Bi/TiO_2_ nano-spheres were prepared using an EISA method [34]. The detailed synthesis procedure can be found in the Experimental section. Briefly, the structure directing agent (F127), Ti (TTIPO), Pt (H_2_PtCl_6_·6H_2_O), and Bi (Bi(NO_3_)_3_·5H_2_O) resources were added into ethanol in sequence, followed by slow evaporation of solvent under mild conditions (40°C, atmospheric pressure) to form a xerogel. After drying at 100°C for 24 h and subsequent calcination in air at 500°C for 6 h, Pt and Bi were anchored onto the TiO_2_ support. The schematic illustration of the synthesis procedure is provided in Fig. 1a. The actual loading amounts of Pt and Bi were determined to be 0.94 wt% and 0.60 wt% (Table 1), respectively, tested by X-ray fluorescence spectroscopy (XRF), which closely matches the nominal values added. Mono-metallic Pt- or Bi-modified TiO_2_ materials were also synthesized for comparison. The resulting materials were referred to as Pt/TiO_2_, Bi/TiO_2_, and Pt-Bi/TiO_2_.

**Table 1.** Physicochemical properties of monometallic and bimetallic Pt- and/or Bi-incorporated TiO_2_ materials.

	Metal loading^a^ (wt. %)			BET surface area^c^ (m^2^·g^−1^)			Pore volume^c^ (cm^3^·g^−1^)		
Samples	Pt	Bi	Pt dispersion^b^ (%)	Micro.	Meso.	Total	Micro.	Meso.	Total
Bi/TiO_2_		0.85		12.4	72.8	85.2	0	0.20	0.20
Pt/TiO_2_	0.87		35.5	23.5	89.2	112.7	0	0.25	0.25
Pt-Bi/TiO_2_	0.94	0.60	79.2	49.8	186.6	236.4	0	0.37	0.37

a The metal loading amount was determined by XRF.

b The Pt metal dispersion was calculated based on H_2_ titration with the detailed experiment method being given in the Experiment part in the online Supplementary information.

c The specific surface area was obtained by the BET method; micropore (micro.) surface area and micropore volume were calculated using the T-Plot method; mesopore (meso.) surface area and mesopore volume were calculated by subtracting the micropore volume from the total volume.

All these TiO_2_-based samples had a crystalline anatase structure (JCPDS no. 21–1272) (Fig. 1b). No discernible diffraction peaks associated with Pt and Bi species were observed on Pt-Bi/TiO_2_, suggesting the uniform dispersion of Pt and Bi species on Pt-Bi/TiO_2_. This conclusion was further supported by Raman spectra analysis (Fig. S1). However, compared to Pt/TiO_2_ and Bi/TiO_2_, Pt-Bi/TiO_2_ showed reduced peak intensity in both XRD and Raman patterns, indicating that the incorporation of Pt and Bi leads to a decrease in the crystallinity of TiO_2_. In addition, a slight shift towards a higher wavenumber was observed in the Raman spectra of the Pt-Bi/TiO_2_ catalyst compared to its monometallic counterparts. This could be due to the partial incorporation of Pt and/or Bi into the TiO_2_ support, where the ionic radius of Pt^4+^ (1.24 Å) and/or Bi^3+^ (1.08 Å) exceeds that of Ti^4+^ (0.68 Å) [35]. These observations illustrate a strong interaction between Pt and/or Bi with the TiO_2_ support, resulting in weakened diffraction peaks and decreased *d*-spacing values of TiO_2_ while preserving its crystalline structure.

The Bi/TiO_2_ composite (Fig. S2) exhibited a distinct partially amorphous connection. Elemental mapping showed the highly homogeneous dispersion of Bi on TiO_2_, without any observable Bi nanoparticles, which strongly indicates the successful doping of Bi into the TiO_2_ lattice. In contrast, the Pt/TiO_2_ composite comprised of irregular nano-spheres (Fig. S3a and b), which clearly demonstrates the presence of Pt nanoparticles (highlighted with red circles). Further analysis using HAADF-STEM (Fig. S3c and d) and elemental mapping images (Fig. S3e–g) revealed the co-existence of numerous large Pt nanoparticles and isolated Pt single atoms/clusters on the TiO_2_ support. Compared to Pt/TiO_2_, the Pt-Bi/TiO_2_ catalyst exhibited pronounced porous features (Fig. S4a). The magnified HRTEM image (Fig. 1c) further elucidates the intricate crystal structure of Pt-Bi/TiO_2_. The interplanar distances were measured to be 0.351 and 0.236 nm, which correspond well with the *d*101 and *d*004 spacing of crystalline anatase TiO_2_ (JCPDS no. 21–1272). The observed larger interplanar distance (*d* = 0.557 nm) is possibly due to Bi or Pt incorporation in the TiO_2_ framework, which induces lattice expansion of the framework. This is consistent with the Raman results, which show a Raman shift after Bi doping. In the HAADF-STEM image (Figs 1d and S4b), Pt metal sites were uniformly dispersed with narrow size distribution and the Pt single-atoms and/or Pt clusters were dominant, contrasting significantly with partially aggregated large Pt nanoparticles observed on Pt/TiO_2_ (Fig. S3c). The compositional line-scan profile (Fig. S4c and d) indicates that the Pt-Bi are distributed individually, with no obvious formation of PtBi alloys. Elemental mapping images further verify that both Pt and Bi, were homogeneously distributed throughout the sample (Fig. 1e).

A typical type IV(a) N_2_ absorption isotherm, characterized by a sharp capillary condensation at high pressures (P/P_0_ = 0.7−0.9), along with an H1-type hysteresis loop, was observed on these two Pt-incorporated materials, indicating the presence of mesopores with cylindrical channels (Fig. 1f) [36,37]. However, Bi/TiO_2_ exhibited a type Ⅱ isotherm and a H4-type hysteresis loop, suggesting an irregular pore structure on this material [36,37]. This observation was further verified by the pore size distribution results (Fig. S5). It is shown that the cylindrical mesopores of Pt/TiO_2_ were radially distributed with a uniform pore size centered around ∼8 nm. Additionally, noticeable mesoporous features with a broad pore size distribution (5–15 nm) were observed for Pt-Bi/TiO_2_. These findings indicate that the addition of Pt precursors (H_2_PtCl_6_·6H_2_O) during the synthesis process promotes the formation of mesopores on Pt/TiO_2_ and Pt-Bi/TiO_2_ catalysts. This phenomenon can be attributed to HCl, which is generated from the decomposition of H_2_PtCl_6_ and acts as a coordination agent, mediating the coordination modes of Ti^4+^ ions and resulting in the formation of partially hydrolyzed [Ti(OH)_n_Cl_m_]^2−^ octahedra (n + m = 6) [38]. The presence of HCl enables effective matching between Ti resource (TTIPO) and Pluronic F127 for cooperative assembly and further facilitates mesopore formation [38].

The textural properties of all materials are summarized in Table 1. Pt-Bi/TiO_2_ had the highest Brunauer–Emmett–Teller (BET) specific surface area (*S*_BET_) of 236.4 m^2^·g^−1^ and the largest mesopore volume (*V*_meso._) of ∼0.37 cm^3^·g^−1^, obtained by subtracting the micropore volume from the total volume. The well-defined mesoporous structure observed on Pt/TiO_2_, as depicted in Fig. 1f, contributed to a higher *S*_BET_ compared to that of Bi/TiO_2_. Further introduction of Pt on Bi/TiO_2_ increased both BET surface area and pore volume. Notably, these TiO_2_-based materials showed significantly higher surface area than commercial P25 (∼50 m^2^·g^−1^) and previously reported mesoporous TiO_2_ [38]. Based on the above analysis, we can conclude that Bi doping in TiO_2_ support can effectively promote the dispersion of Pt species and induce a high ratio of Pt single atoms/clusters, as shown in Fig. 1g.

### Electronic structure characterization and analysis

XPS was employed to study the interactions between Pt and the support. All three samples exhibited a resonance at 529.8 eV in the O 1s spectrum (Fig. S6a), corresponding to the lattice oxygen species (Ti-O, Bi-O, and Pt-O) [39–41]. Meanwhile, a shoulder peak at ∼531.5 eV was observed, indicative of hydroxyl groups on the surface of TiO_2_ [42], as well as the surface adsorbed oxygen species (O_2_^−^, and/or O^−^, O_ads._) induced by oxygen vacancies (OVs) [39]. The Ti 2p spectra (Fig. S6b) showed two main peaks at ∼458.6 and 464.3 eV, representing the Ti(IV) valence state on lattice oxygen [43]. The resonance at ∼457.0 and 462.9 eV, attributed to Ti(III) [44] were rarely observed on these samples. Nevertheless, Ti(III) content was observed to increase from ∼0.03% on Bi/TiO_2_ to ∼0.05% on Pt-Bi/TiO_2_ (Table S1), suggesting a concurrent increase in lattice OVs over Pt-Bi/TiO_2_, which is consistent with the XPS result of O 1s.

**Figure 2.**
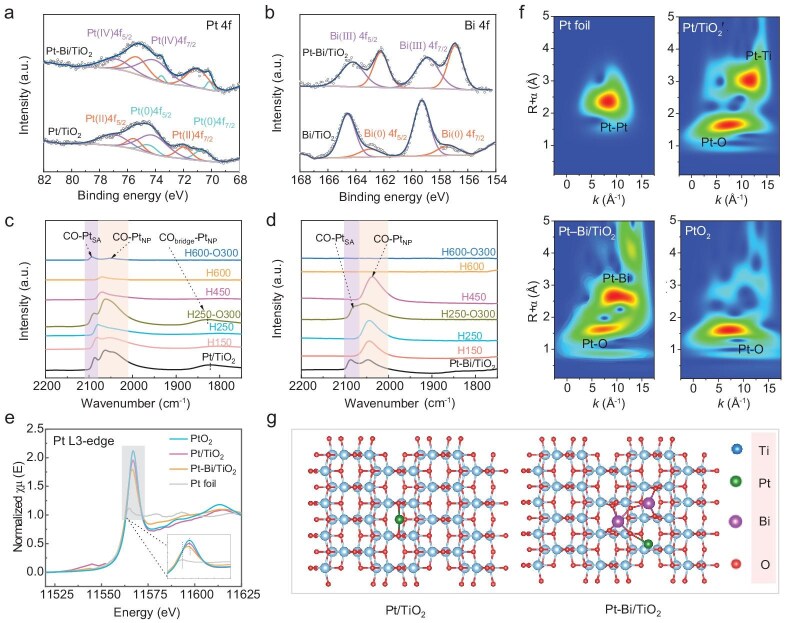
(a and b) XPS spectra of Bi/TiO_2_, Pt/TiO_2_ and Pt-Bi/TiO_2_ samples: (a) Pt 4f spectra and (b) Bi 4f spectra. CO-DRIFT spectra of (c) Pt/TiO_2_-H_x_-O_y_ and (d) Pt-Bi/TiO_2_-H_x_-O_y_ showing CO adsorption after Ar purging at room temperature. The shaded background refers to different CO-adsorbed Pt sites. Pt L3-edge XAS spectra: (e) normalized Pt L3-edge XANES and (f) wavelet transform (WT) for the k3-weighted Pt L3-edge EXAFS. For comparison, the XAS spectra of standard Pt foil and PtO_2_ are also included. (g) The structure scheme of Pt/TiO_2_ and Pt-Bi/TiO_2_.

The deconvoluted XPS spectra of Pt 4f and Bi 4f orbital levels were analyzed (Fig. 2a and 2b), and the surface proportions of Pt and Bi at different chemical states were calculated (Tables S2 and S3). On both Pt/TiO_2_ and Pt-Bi/TiO_2_ catalysts, three Pt resonances located at ∼70–80 eV were observed, which can be attributed to Pt(0), Pt(II), and Pt(IV) species (Fig. 2a) [45,46]. The two spin-orbital components of 4f_7/2_ and 4f_5/2_ at ∼70.5 and 73.5 eV exhibited an asymmetric shape on Pt/TiO_2_, which is characteristic of metallic platinum (Pt(0)). The formation of Pt(0) could be due to the decomposition of PtO_2_ to PtO and subsequently to metallic Pt during high-temperature calcination [47]. The resonances of the platinum oxide doublets (Pt(II) and Pt(IV)) were also observed over two samples. After Bi doping, a slightly downward shift in binding energies of Pt(0), Pt(II), and Pt(IV) was observed (Table S2 and Fig. 2a). The observed shifts in binding energies indicate a stronger interaction between Pt metal species and the Bi/TiO_2_ support on the Pt-Bi/TiO_2_ catalyst than the Pt/TiO_2_ catalyst [47].

Both the oxidative and metallic Bi species were detected on Bi/TiO_2_ and Pt-Bi/TiO_2_ samples, evidenced by the typical characteristic doublets of Bi 4f7/2 and Bi 4f5/2 (Table S3 and Fig. 2b), respectively. Likewise, the binding energies of Bi species on Pt-Bi/TiO_2_ also shifted downwards compared to Bi/TiO_2_, indicating the strong metal-support interaction between Bi and support as well. Moreover, the proportion of Pt and Bi species with different states (Tables S2 and S3) revealed that the increased fraction of surface oxidative Pt species (Pt^δ+^) was accompanied by a decrease in Bi(III) species on the bimetallic catalyst, clearly illustrating electron transfer between Pt, Bi, and support on Pt-Bi/TiO_2_. The Bi doping hinders the self-reduction of Pt, resulting in the formation of more Pt^δ+^ species.

The presence of OVs was investigated by EPR spectroscopy. In comparison to the Pt/TiO_2_ and Bi/TiO_2_ samples, a significantly enhanced EPR signal centered at g = 2.0044 was observed on the Pt-Bi/TiO_2_ catalyst (Fig. S7), a characteristic feature associated with unpaired electrons that are localized within OVs [48,49]. Such enhancement in the EPR signal indicates that Bi doping benefits the formation of abundant OVs. The existence of abundant OVs promotes efficient electron transfer between metals and the support, resulting in an elevated density of localized unpaired electrons and facilitating the redistribution of extra electrons to adjacent metal atoms, Additionally, an extended light absorption detected by UV-Vis diffuse reflectance spectroscopy (UV/Vis DRS) implies the increased absorption of visible light on the bimetallic Pt-Bi/TiO_2_ catalyst (Fig. S8a). The observed slight red shift after Bi doping can be attributed to electron transitions caused by abundant Ti(III) species, OVs, and other defects at different energy levels, as mentioned above [38]. Besides, compared to bulk TiO_2_, which exhibited an absorption edge at ∼400 nm [38], both monometallic Bi/TiO_2_ and Pt/TiO_2_ demonstrated a significant enhancement in light absorption within the visible light range. Band gap calculations (Fig. S8b) reveal that the Pt-Bi/TiO_2_ catalysts exhibited a narrower band gap (2.75 eV), which facilitates easier electron transitions from the valence to the conduction band, thereby enhancing intrinsic carrier concentration, conductivity, and overall activity. The enhanced electron mobility was also verified by the H_2_ temperature-programmed reduction (H_2_-TPR) analysis (see Fig. S9 and the detailed discussion in the online Supplementary file).

*In situ* DRIFT spectroscopy using CO as the probe molecular was employed to uncover the metal-support interactions over the Pt-Bi/TiO_2_ catalysts. Three samples (Pt/TiO_2_, Bi/TiO_2_, and Pt-Bi/TiO_2_) were reduced or oxidized at different temperatures and denoted as sample-H_x_ and sample-H_x_-O_y_, where x and y represent the temperature of reduction or oxidation. Fig. 2c and 2d depict the DRIFT spectra of CO adsorption on Pt/TiO_2_, and Pt-Bi/TiO_2_ under various treatment conditions. Three main bands centered at 2086, 2059, and 1830 cm^−1^ were observed on the Pt/TiO_2_ catalyst (Fig. 2c). In contrast, no CO adsorption was detected on the Bi/TiO_2_ sample under identical experimental conditions (Fig. S10), indicating that these peaks originate solely from CO molecules adsorbed on Pt species. The band at 2086 cm^−1^, which remained unshifted with changes in CO coverage (Figs S11–S14), is associated with CO adsorption on Pt SAs (CO-Pt_SA_) [50–52]. Conversely, the bands at 2059 and 1830 cm^−1^ correspond to linearly and bridgedly adsorbed CO on Pt nanoparticles (CO-Pt_NP_) [50]. The shifts of the peak at 2059 cm^−1^ during desorption after argon (Ar) purge (Figs S11 and S13) are correlated with the change in dipole-dipole coupling between CO molecules on those Pt NPs [51]. For Pt/TiO_2_, under low reduction temperatures (150–250°C), CO adsorption on Pt SAs was well preserved with a negligible peak shift (Figs 2c and S12a). Only when the reduction temperature reached 450°C, did the CO adsorption on Pt SAs disappear completely, indicating the strong metal-support interaction (SMSI) between Pt SAs and the support only at high reduction temperatures [53]. The reversibility of CO adsorption on Pt SAs could be because SMSI results in electron transfer from partially reduced oxide cations, Ti^3+^, to adjacent Pt metal atoms at the oxide–metal interface, giving rise to negatively charged metal species (Pt^δ−^) [54–59]. Furthermore, driven by SMSI, partially reduced oxide layers tend to migrate onto the metal surface, commonly leading to the formation of partial or complete encapsulation structures [54–59]. Therefore, no CO absorption was detected. However, subsequent re-oxidation at 300°C restored the CO-Pt_SA_ peak, demonstrating the encapsulation reversibility of Pt SAs by reduced TiO_2_ oxide layers and ruling out the possibility of their aggregation into larger particles [50]. In contrast, CO adsorption on Pt NPs was retained even after reduction at 600°C, indicating a weaker interaction between Pt NPs and support than that of Pt SAs. After two reduction-oxidation cycles, both CO-Pt_SA_ and CO-Pt_NP_ over Pt/TiO_2_ emerged, unambiguously illustrating the reversible incorporation of Pt SAs and NPs in TiO_2_.

Interestingly, the reduction treatment at 150°C resulted in the complete disappearance of CO-Pt_SA_ over the Pt-Bi/TiO_2_ catalyst (Figs 2d and S14). This is most probably attributed to the fast electron transfer between Pt SAs and Bi/TiO_2_, leading to pronounced SMSI between Pt SAs and support at low reduction temperatures (150°C) [50]. Likewise, the CO-Pt_SA_ can be regenerated after subsequent re-oxidation at 300°C. As anticipated, the CO-Pt_NP_ peaks were retained even after reduction at 450°C. Upon reduction at 600°C, all peaks vanished irreversibly, suggesting the enhanced interaction between Pt species and the support after the doping of Bi into the catalyst. These findings jointly reveal that Pt-Bi/TiO_2_ has more oxygen vacancies than Pt/TiO_2_, which could promote SMSI and the electron transfer between Pt species to Bi/TiO_2_ support.

Furthermore, the X-ray absorption near edge structure (XANES) spectra of the Pt L3-edge, which are sensitive to the valence states of Pt, are provided in Fig. 2e. Both Pt/TiO_2_ and Pt-Bi/TiO_2_ exhibited a strong white line peak close to that of PtO_2_, indicating their highly oxidized states of Pt species in the as-prepared Pt-based catalyst [60]. Upon zooming in, as shown in the inset, it is found that the absorption edge positions were slightly shifted towards the lower energy range, compared with that of the standard PtO_2_ sample. This confirms their relatively lower average Pt valences than PtO_2_, consistent with the XPS results above. The Fourier transform (FT) k3-weighted extended X-ray absorption fine structure (EXAFS) spectra in *R* and *k* space of these two samples and the fitted spectra are provided in Fig. S15. Compared to the Pt foil, as shown in *R* space in Fig. S15c, the peak Pt−Pt path was not present in the Pt/TiO_2_ and Pt-Bi/TiO_2_, further confirming the single-atom Pt species dominantly presented on both samples. Meanwhile, a predominant peak at ∼*R* = 1.65 Å was observed for Pt/TiO_2_, Pt-Bi/TiO_2_ and PtO_2_. This path belonging to the first-shell Pt-O coordination confirms that Pt atoms are coordinated with O in both Pt/TiO_2_ and Pt-Bi/TiO_2_. It is noteworthy that both Pt/TiO_2_ and Pt-Bi/TiO_2_ also showed additional peaks between 2 and 3 Å which were not remarkable in that of Pt metal and PtO_2_, suggesting that the local structures of Pt species within these two samples become quite different. According to the fitting results (Fig. S15d and Table S4), these additional second-shell paths are attributed as Pt-(O)-Ti and Pt-(O)-Bi, suggesting the involvement of Ti and Bi in the local structures of Pt species in the Pt/TiO_2_ and Pt-Bi/TiO_2_. Due to the formation of Pt-O-Bi structure after Bi doping, the Pt−O path length of Pt-Bi/TiO_2_, noted as R in the table (R ≈ 1.99 Å, N ≈ 0.99), was larger than that of Pt/TiO_2_ (R ≈ 1.97 Å, N ≈ 2) [61]. Moreover, the coordination number (N) of first-shell Pt-O was decreased in Pt-Bi/TiO_2_, indicating abundant OVs existence after Bi doping, in accordance with the results discussed above. These results well confirm the isolated single atomic dispersion of Pt on both samples, which was also supported by EXAFS wavelet transformation (WT) analysis (Fig. 2f). Pt/TiO_2_ showed one well-defined circular intensity maximum with the predominance of Pt-O, in contrast to the early moon shape observed in Pt-Bi/TiO_2_, indicating that the local structure of Pt is different from Pt-Bi/TiO_2_ due to the addition and the coordination of Bi. Notably, both of them showed the intensity between 2 and 3 Å localized in a different *k* range from Pt-Pt coordination (10–15 Å^−1^ compared to 5–10 Å^−1^), consistently implying that the EXAFS peak around 2.2 Å (Fig. S15c) was attributed to the coordination of Pt with Ti or Bi [62]. The observation of Pt-(O)-Ti indicates that unsaturated coordinated Ti(Ⅲ) near OVs bond with Pt atoms, supporting the OVs anchoring mechanism for Pt single-atoms.

DFT calculations were used to model the details of the local structure of Pt and Bi on the TiO_2_ support based on these XAS results (Fig. 2g). Based on the XAS results and DFT-optimized structures, the most energetically and structurally favorable configuration was identified, in which Pt single-atoms are anchored at the hollow sites of the (101) surface of nanosized TiO_2_ (Fig. 2g). This structure provides the best match to the combined experimental data.

### Base-free catalytic oxidation of biomass-derived HMF

**Figure 3.**
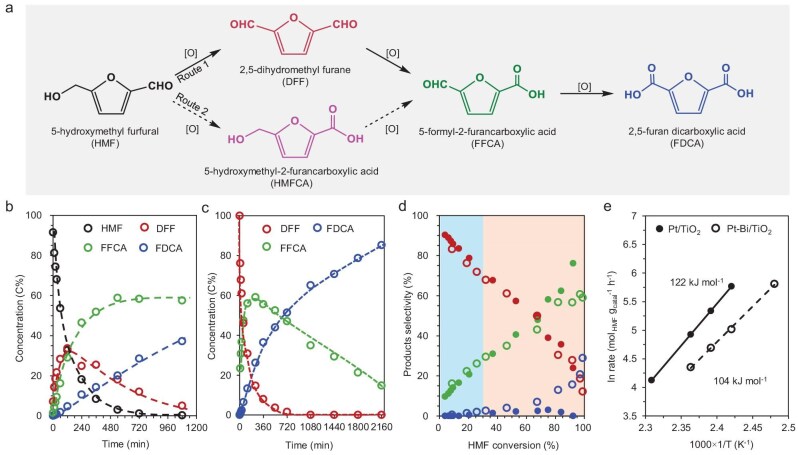
(a) The scheme of HMF oxidation process; carbon-based concentration-time profiles of (b) HMF and (c) DFF oxidation on Pt-Bi/TiO_2_ catalyst; (d) the main products selectivity as a function of HMF conversion and (e) Arrhenius plots based on the initial rate of HMF oxidation over Pt/TiO_2_ and Pt-Bi/TiO_2_. Solid symbol in (d) is for Pt/TiO_2_, and hollow symbol for Pt-Bi/TiO_2_; red circles, DFF; green circles, FFCA; blue circles, FDCA. Reaction conditions: 100 mg catalyst, 80 mL 0.03 M HMF aqueous solution, 3 MPa (ambient temperature) air, stirred at 700 rpm, 150°C. The reaction results over Pt/TiO_2_ and Bi/TiO_2_ are shown in Figs S16–S18.

The HMF oxidation reaction was conducted under mild conditions (T = 150°C, P_air_ = 3 MPa) over Bi/TiO_2_, Pt/TiO_2_ and Pt-Bi/TiO_2_ catalysts. The proposed reaction routes for the conversion of HMF to FDCA are shown in Fig. 3a. The oxidation of HMF to FDCA is a multistep process involving the activation of various functional groups, resulting in the formation of a range of furanyl compounds, including DFF, HMFCA, FFCA, and FDCA [63,64]. The carbon-based concentrations of HMF and the major products as a function of reaction time over Pt/TiO_2_ and Pt-Bi/TiO_2_ are shown in Figs S16b and S3b. At the initial stage of the reaction, DFF—produced via oxidation of the hydroxymethyl group in HMF—was identified as the major product. Sequential oxidation then led to the formation of FFCA and ultimately FDCA. Notably, HMFCA was not observed in our study, suggesting HMF→DFF→FFCA→FDCA is the major reaction pathway over the studied catalysts. Previous studies have indicated that the aldehyde group of HMF oxidizes more easily to form HMFCA under alkaline conditions, particularly in the presence of noble metal catalysts, such as Ag [65], Pt [6], Ru [11], and their alloys [66] due to a lower reaction energy barrier. In contrast, under base-free aqueous conditions, DFF is preferentially formed as a key intermediate [67] which aligns with our experimental results. As depicted in Fig. S16b, Pt/TiO_2_ afforded only minimal production of FDCA (∼1.8%) even at ∼80% HMF conversion. The initial conversion rate (represented by turnover frequency (TOF)) of HMF was determined to be 156.5 h^−1^ (see the detailed calculation method given in the Experimental part of the online Supplementary information).

In contrast, the complete conversion of HMF (>99%, at 720 min) was achieved over Pt-Bi/TiO_2_ catalyst under identical reaction conditions with a significantly higher initial rate of 367.9 h^−1^ (Fig. 3b). Interestingly, when HMF conversion was below 30% (Fig. 3d), the selectivity towards DFF, FFCA, and FDCA remained comparable regardless of Bi doping. However, upon exceeding 30% conversion of HMF, Pt-Bi/TiO_2_ exhibited notably higher selectivity to FDCA. It is worth noting that almost no activity was observed over Bi/TiO_2_ (Fig. S17), suggesting that Pt species serve as the catalytic active sites for the oxidation of HMF while Bi species act as promoters to not only accelerate the reaction rates but also to strengthen the formation of FDCA. The much higher selectivity towards FDCA upon exceeding 30% conversion of HMF over Pt-Bi/TiO_2_ might be caused by the enhanced competitive adsorption capability of FFCA compared to Pt/TiO_2_ in the presence of high concentrations of HMF or the improved catalytic activity for the cascade steps of DFF→FFCA→FDCA over bimetallic catalysts.

To validate our presumption, oxidation of DFF under the same reaction conditions was conducted over the Pt/TiO_2_ and Pt-Bi/TiO_2_ catalysts (Figs S16c and S3c). Similar to the HMF oxidation, the conversion of DFF on Bi/TiO_2_ was negligible (∼15% conversion at 360 min, see Fig. S18). By contrast, Pt/TiO_2_ and Pt-Bi/TiO_2_ exhibited superior catalytic activity in DFF conversion, with initial conversion rates of 254.1 and 461.3 h^−1^, respectively. This suggests that the inappreciable formation of FDCA over Pt/TiO_2_ when HMF is the substrate is primarily caused by the limited adsorption of FFCA in the presence of HMF rather than its low reaction rates of DFF. Only at low HMF concentrations with conversion >80%, did the selectivity towards FDCA increase. This conclusion was further validated by an adsorption experiment of FFCA over Pt/TiO_2_ and Pt-Bi/TiO_2_ catalysts in the presence of equimolar amounts of either HMF or DFF, as shown in Fig. S19. The results indicate that FFCA exhibited negligible adsorption on both Pt/TiO_2_ and Pt-Bi/TiO_2_ when mixed with HMF. Furthermore, the enhanced adsorption capacity of the Bi-doped catalyst in the FFCA and HMF mixture was also observed. In contrast, substantial adsorption of FFCA (>40% adsorbed under ambient temperature) was observed on both catalysts when mixed with DFF. This observation confirms that the adsorption of FFCA on the catalyst surface influences the yield of FDCA. The high adsorption capacity of catalysts towards FFCA promotes further conversion of FFCA to FDCA. In conclusion, Bi doping facilitates the absorption of FFCA, further promoting the conversion of FFCA to FDCA in the HMF oxidation reaction, although the overall FDCA yield still remained low. Besides, unlike the reaction of HMF (Fig. 3d), the almost identical selectivity to FFCA and FDCA at the comparable conversion levels of DFF over both Pt/TiO_2_ and Pt-Bi/TiO_2_ catalysts (Fig. S16d) revealed that Bi doping exclusively accelerates the oxidation of -CHO groups to -COOH.

To further verify the promoting effect of Bi on the oxidation of HMF, a kinetic study was performed with varying reaction temperatures (140–160°C for Pt/TiO_2_ and 130–150°C for Pt-Bi/TiO_2_, see Fig. S20); the apparent activation energy for HMF oxidation over Pt/TiO_2_ and Pt-Bi/TiO_2_ was determined to be 122 and 104 kJ·mol^−1^ (Fig. 3e), respectively. These results demonstrate that doping Bi reduced the activation barrier of HMF conversion, consequently leading to much higher reaction rates [68]. The decrease in the activation barrier could be ascribed to the facilitated adsorption and activation of oxygen-containing functional groups and molecular O_2_ triggered by the abundant surface OVs, as well as the enhanced electron transfer between Pt^δ+^ species and surface reactive intermediates over the Pt-Bi/TiO_2_ catalyst [69,70]. The carbon molar balance for the oxidation of HMF and DFF was evaluated using Bi/TiO_2_, Pt/TiO_2_, and Pt-Bi/TiO_2_ catalysts (Fig. S21). The analysis considered all identified reaction products in the liquid phase, along with unreacted substrates, after 4 h of reaction. In addition, gas-phase products were detected (Fig. S22), with CO_2_ identified as the main component. The unaccounted portion of the carbon balance is likely due to gaseous products or undetected reaction intermediates.

In the case of HMF oxidation, >90% of the carbon was recovered as liquid-phase products when using Pt/TiO_2_ and Pt-Bi/TiO_2_, indicating high selectivity and conversion efficiency (Fig. S21). In contrast, the Bi/TiO_2_ catalyst showed a significantly lower yield of liquid products, which may result from the formation of polymeric or overoxidized byproducts. For DFF oxidation, all three catalysts exhibited a high carbon balance in the liquid phase, suggesting minimal formation of gaseous or undetectable intermediates. These results highlight the critical role of Pt as the primary active site for facilitating the oxidation of HMF to DFF. DFT calculations were subsequently employed to disclose the underlying promotion effects of Bi over Pt-Bi/TiO_2_ on the ‘base-free’ catalytic oxidation of HMF.

### DFT calculation analysis

**Figure 4.**
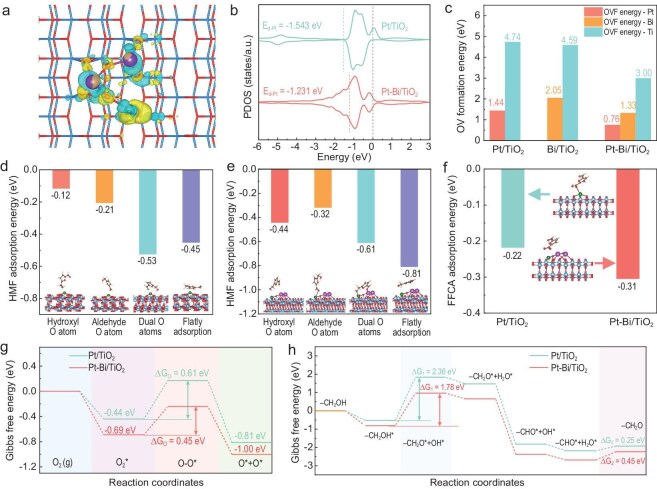
(a) Charge density difference plot on the surface of Pt-Bi/TiO_2_. Yellow and blue respectively represent an increase and decrease in electron cloud density, with an interface value of 0.005 e·bohr^−3^. The TiO_2_ support is illustrated in a stick model, with Bi atoms shown as purple spheres and Pt atoms as green spheres. (b) Projected density of states (PDOS) of Pt/TiO_2_ and Pt-Bi/TiO_2_. (c) OVs formation energies on Bi, Pt, and Ti; HMF adsorption energies of different adsorption states on (d) Pt/TiO_2_ and (e) Pt-Bi/TiO_2_. (f) FFCA adsorption energies on Pt/TiO_2_ and Pt-Bi/TiO_2_. (g) Change of Gibbs free energy during O_2_ dissociation, and (h) change of Gibbs free energy during HMF initial oxidation to DFF over Pt/TiO_2_ and Pt-Bi/TiO_2_.

After confirming that the single atomic Pt species are the main active species on Pt/TiO_2_ and Pt-Bi/TiO_2_ catalysts, the structures of Pt/TiO_2_, Bi/TiO_2_ and Pt-Bi/TiO_2_ were constructed according to the results of XAS analysis and DFT calculation. The charge density difference on Pt/TiO_2_ revealed a noticeable decrease in the electron density cloud on the Pt surface, whereas an increase was observed on the Ti surface (Fig. S23a), indicating charge transfer from Pt species to TiO_2_. Moreover, for Bi/TiO_2_, an SMSI between Bi and TiO_2_ was observed due to the formation of a heterojunction (Fig. S23b). Upon formation of the triphasic Bi-Pt-TiO_2_ heterojunction structure, the surface-deposited Pt demonstrated enhanced interfacial interactions with both Bi and TiO_2_ (Fig. 4a). Also, the electron localization function (ELF) was used to observe the surface charge distribution of TiO_2_ after Pt, Bi, and Pt-Bi incorporation (Fig. S24). For mono-metallic catalysts, Pt or Bi formed stable chemical bonds with surface O on TiO_2_ and TiO_2_, respectively. However, for Pt-Bi/TiO_2_, Pt species were located near an OV. Density of states (DOS) (Fig. S25) and projected density of states (PDOS) analysis (Figs 4b and S26) further verified the enhanced electron transfer between Pt species and Bi/TiO_2_ after Bi doping. Due to the uniform dispersion characteristic of Pt and/or Bi species on TiO_2_ support as discussed above, the DOS of all samples exhibited a similar energy band distribution compared to the TiO_2_ support near the Fermi level (Fig. S25). Moreover, compared to that on Pt/TiO_2_ (−1.543 eV), the *d*-band of Pt (−1.231 eV) on Pt-Bi/TiO_2_ exhibited a greater shift close to the Fermi level (Fig. 4b), indicating an increase in surface charge of Pt. Bader charge analysis quantified the SMSI effect and electron transfer capability between Pt species and the support (Fig. S27 and Table S5). The higher Bader charge number of Pt species in Pt-Bi/TiO_2_ accurately reflected the relatively lower coordination state of Pt species than that over Pt/TiO_2_ and the enhanced electron transfer between Pt/Bi species and the TiO_2_ support. These conclusions are consistent with the obtained results from XPS, *in situ* DRIFTs, and XAS characterizations as discussed above.

Further analysis was conducted to investigate the state of surface oxygen species. First, the positions and formation energies of OVs on Bi/TiO_2_, Pt/TiO_2_, and Pt-Bi/TiO_2_ were calculated (Figs S28 and 4c). It is observed that generating OVs on the Pt and Bi sites was comparatively easier than that on TiO_2_. In addition, Pt-Bi/TiO_2_ exhibited lower oxygen vacancy formation (OVF) energy on both Pt/Bi sites and TiO_2_ than those on Bi/TiO_2_ and Pt/TiO_2_, rendering it more prone to forming OVs. This is consistent with the EPR results as discussed above, where Pt-Bi/TiO_2_ was conducive to generate more OVs.

The surface adsorption states of HMF on these catalysts were subsequently simulated (Figs 4d, 4e, and S29a). On the Pt/TiO_2_ surface (Fig. 4d), steric hindrance promoted bridge adsorption (−0.53 eV) and flat adsorption (−0.45 eV) of HMF. Similarly, on the Pt-Bi/TiO_2_ surface (Fig. 4e), both bridge adsorption (−0.81 eV) and flat adsorption (−0.61 eV) of HMF were readily achieved. The respective adsorption energies of HMF on these catalysts reveal that the presence of smaller Pt species and the synergistic effect between Pt and Bi enhance the adsorption of HMF on the Pt-Bi/TiO_2_ surface. Further adsorption energy calculations of FFCA on these catalysts (Figs 4f and S29b) show that FFCA is more prone to be absorbed on Pt-Bi/TiO_2_ than Pt/TiO_2_ and Bi/TiO_2_ (−0.31 eV vs −0.22 eV and −0.07 eV). However, compared to HMF, FFCA is more difficult to adsorb on both catalysts, which could further inhibit the reaction of FFCA towards the formation of FDCA at high concentrations of HMF. This is correlated with the results of experiments as discussed above. Additionally, the structure of HMF and the corresponding structure parameters of bonds after surface adsorption are shown in Fig. S30 and Table S6, respectively. Compared to the H1-O1 bond and C1-O1 bond lengths of HMF on Pt-Bi/TiO_2_ under flat adsorption (0.9765 Å, 1.4190 Å) and aldehyde adsorption (0.9746 Å, 1.4332 Å), the dual adsorption (0.9850 Å, 1.4461 Å) and hydroxyl adsorption (0.9836 Å, 1.4456 Å) models of HMF on Pt-Bi/TiO_2_ displayed longer bond lengths. It is evident that dual adsorption and hydroxyl adsorption models of HMF on Pt-Bi/TiO_2_ resulted in better activation of the H1-O1 bond and C1-O1 bond compared to Pt/TiO_2_, facilitating the oxidative removal of -CH_2_OH.

DFT calculations further revealed the adsorption and dissociation models of oxygen on both Bi/TiO_2_, Pt/TiO_2_ and Pt-Bi/TiO_2_ (Fig. S31), weak chemical adsorption of oxygen with significant different adsorption capacities and dissociation energy at these materials’ interfaces (Figs S29c and 4g). Pt-Bi/TiO_2_ (∆G = 0.69 eV) demonstrated a higher propensity for activating oxygen molecules into active oxygen species than Pt/TiO_2_ (∆G = 0.44 eV) and Bi/TiO_2_ (∆G = 0.27 eV). This may also be attributed to the abundant OVs on Pt-Bi/TiO_2_ compared to Pt/TiO_2_. The SMSI enhances the ability of Pt sites to activate oxygen molecules and generate reactive oxygen species when compared to Pt/TiO_2_, which greatly promotes the oxidation of HMF and DFF.

The free energy change in the oxidation of HMF to DFF, which is the initial step in the whole reaction course of HMF→DFF→FFCA→FDCA, was calculated (Figs S29d and 4h) on Bi/TiO_2_, Pt/TiO_2_, and Pt-Bi/TiO_2_ surfaces based on the proposed elementary steps depicted in Scheme S1 [71,72]. The energy required for removal of the first H from the -CH_2_OH group of HMF via the breaking of the O-H bond as a rate-determining step was lower on Pt-Bi/TiO_2_ (∆G_1_ = 1.78 eV) than that on the Pt/TiO_2_ (∆G_1_ = 2.36 eV) and Bi/TiO_2_ (∆G_1_ = 3.05 eV) surface. After breaking O-H, the resultant unsaturated coordinated -CH_2_O* can easily undergo further oxidative breakdown to form the -CHO group to remove DFF product from the surface of the catalysts.

## CONCLUSIONS

In summary, we have successfully developed a facile method for synthesizing Pt single-atoms dominantly supported on a Bi-doped mesoporous TiO_2_ catalyst, enabling the efficient oxidation of biomass-derived HMF to high-value-added fine chemicals under ‘base-free’ conditions. Compared to the monometallic Pt/TiO_2_, the Pt-Bi/TiO_2_ catalyst demonstrated markedly enhanced metal-support interactions, a reduced Pt metal size, an increased surface area of the catalyst, and a higher density of oxygen vacancies. Further reaction kinetic study and DFT calculations elucidated the function of Pt and Bi, as well as their synergistic effects on the catalytic oxidation of HMF. The changes in geometric and electronic structures induced by the doping of Bi over Pt-Bi/TiO_2_ effectively lowered the dissociation energy of O_2_ into active oxygen species, reduced the adsorption energy of HMF as well as the activation barrier for its conversion, collectively accelerating the conversion reaction rate of HMF. Overall, the present research offers in-depth understanding of the complex interplay among different components on the Pt-Bi/TiO_2_ catalyst and their collective influence on catalytic oxidation reactions. These findings provide convincing evidence for the enhanced catalytic activity achieved by incorporating both Pt and Bi metals into the TiO_2_ support, highlighting great potential for catalyst design in ‘base-free’ aerobic oxidation of HMF to value-added chemicals using environmentally benign water as the solvent.

## Supplementary Material

nwaf327_Supplemental_Files
