# Supplementary material for: Strong metal-support interaction induced by Pt-O-Bi bonding in mesoporous anatase TiO2 for base-free catalytic biomass valorization
Source: Natl Sci Rev. 2025 Aug 26;12(10):nwaf327. doi: 10.1093/nsr/nwaf327 (PMC12491999; doi:10.1093/nsr/nwaf327)
Supplement: nwaf327_Supplemental_Files [file nwaf327_supplemental_files.zip › Supplementary data.pdf]

SUPPLEMENTARY INFORMATION

Strong metal-support interaction induced by Pt-O-Bi bonding in mesoporous anatase TiO_2 for base-free catalytic biomass valorisation

Haimei Xu[1,2,#], Chao Feng[1,#], Yunzhao Fan[1,#], Huawei Geng[1], Qisong Yi[1,4], Xiaoning Li[2,3], Yameng Fan[2,3], Yibo Ma[2,3], Baohua Jia[2,3], Yuanshuai Liu[1,*], Valentin Valtchev [5,*] and Tianyi Ma[2,3,*]

[1]Qingdao Institute of Bioenergy and Bioprocess Technology, Chinese Academy of Sciences, Qingdao 266101, China;

[2]Centre for Atomaterials and Nanomanufacturing (CAN), School of Science, RMIT University, Melbourne, VIC 3000, Australia;

[3]ARC Industrial Transformation Research Hub for Intelligent Energy Efficiency in Future Protected Cropping (E2Crop), Melbourne, VIC 3000, Australia;

[4]University of Chinese Academy of Sciences, Beijing 100049, China;

[5]Normandie Univ, ENSICAEN, UNICAEN, CNRS, Laboratoire Catalyse et Spectrochimie, Caen 14050, France

***Corresponding authors.** E-mails: liuys@qibebt.ac.cn; valentin.valtchev@ensicaen.fr; tianyi.ma@rmit.edu.au

[#]Equally contributed to this work.

METHOD

Materials

All chemicals employed were of analytical grade and used without any further modification. These chemicals include poly(ethylene glycol)-b-(propylene glycol)-b-(ethylene glycol) (Pluronic F127, average molecular weight of 12600, Sigma-Aldrich), anhydrous ethanol (Sinopharm, 99.5%), titanium (IV) isopropoxide (TTIPO, 97%, Sigma-Aldrich), chloroplatinic acid hexahydrate ($H_2PtCl_6 \cdot 6H_2O$, ≥37.50% Pt basis, Sigma-Aldrich) bismuth (III) nitrate pentahydrate ($Bi(NO_3)_3 \cdot 5H_2O$, ≥98.0%, Sigma-Aldrich) and hydrochloric acid (HCl, Sinopharm, 36%~38%, Sinopharm).

Catalyst fabrication

Bimetallic Pt supported Bi-doped mesoporous TiO_2 catalyst, denoted as Pt-Bi/TiO_2, was prepared via a one-pot evaporation-induced self-assembly (EISA) method [1]. In a typical procedure, 1.5 g of Pluronic F127 was first suspended in 20 mL of anhydrous ethanol, followed by stirring for 0.5 h at ambient temperature. Next, a nominal weight ratio of TTIPO, $H_2PtCl_6 \cdot 6H_2O$, and $Bi(NO_3)_3 \cdot 5H_2O$, were successively added to the above suspension under vigorous stirring overnight. The mixture was subsequently transferred into the oven and subjected to a temperature of 40℃ for 48 h, followed by an additional reaction period at 100℃ for 24 h. Finally, the obtained transparent xerogel was calcined in a static air environment at 500℃ for 6 h. The loading of Pt and Bi, based on the weight of TiO_2 were 1 and 0.6 wt%, respectively. For comparison, monometallic Pt/TiO_2 and Bi/TiO_2 catalysts with the same metal loadings were also synthesized using identical method and using HCl to adjust the pH being same with that during the synthesis of Pt-Bi/TiO_2.

Textual and electronic structure characterizations

The metal loading of all samples was determined by X-ray fluorescence spectrometer (XRF) on a ZSX Primus instrument. Powder X-ray diffraction (XRD) patterns were recorded on a PANalytical X'Pert PRO Multipurpose Diffractometer at 40 kV voltage and 20 mA current using Cu Kα radiation (λ=1.5406 Å). Raman spectra were collected using a confocal LabRAM HR Evolution instrument (Horiba) equipped with a visible 633 nm laser and a high-grade Leica microscope. Nitrogen adsorption/desorption isotherms were measured on a Quantachrome Autosorb iQ at 77 K after overnight degassing of the samples under vacuum at 120℃. High-resolution transmission electron microscopy (HRTEM) was recorded on a JEOL 2100 TEM operated at a voltage of 200 kV. High-angle annular dark field-scanning transmission electron microscopy (HAADF-STEM) images were acquired using a FEI Themis-Z Double-corrected 60-300 kV STEM operated at 300 kV. The convergence and collection angle under the HAADF-STEM mode is 17.9 mrad and 50-200 mrad, respectively. The point resolution of Themis-Z under the STEM mode is around 0.6 Å (operated at 300 kV). Atomic resolution elemental mapping data was obtained by combining the HAADF-STEM imaging mode with EDS.

The H_2 temperature-programmed reduction (H_2-TPR) and H_2-O_2 titration were carried out on a Micromeritics AutoChem Ⅱ instrument with a thermal conductivity detector (TCD). For H_2-TPR measurement, samples (50 mg) were first pretreated with pure Ar at 350℃ for 1 h (75 mL/min). After cooling to room temperature and purging with a reducing gas mixture of 5% H_2/N_2 (75 mL/min), the H_2-TPR was performed by increasing the temperature from 50 to 800℃ at a heating rate of 10 ℃/min. H_2 consumption was recorded using a TCD detector. For the H2 titration experiment, 50 mg sample was first pretreated under 350℃ with He for 1 h and then reduced under

500℃ with 5% H_2/N_2 for 1 h. After purged with pure N_2 for another 1h until the temperature down to 100℃, activated Pt species on the surface of the catalyst was titrated by H_2 pulses until the equal areas of eluted peaks were observed. The Pt dispersion (D) was calculated from the volume of H_2 titrated by the following equation:

$$D=2VM*100/(22414*W*P)$$

Where V is the volume of H_2 used (mL), W is the catalyst mass (g), P is the Pt mass fraction in the tested catalyst (%), M is the atomic mass of Pt (g/mol).

X-ray photoelectron spectra (XPS) were recorded an AXIS SUPRA spectrometer (Kratos). The binding energy of adventitious carbon (C 1s) at 284.8 eV was used to correct the charging effect of samples. Ultraviolet-visible (UV–vis) diffuse reflectance spectra were obtained on a Shimadzu spectrometer, using $BaSO_4$ as blank. Electron paramagnetic resonance (EPR) spectra was recorded at room temperature on a Bruker A-200 spectrometer operated at 9.78 GHz. Hard X-ray absorption spectroscopy (XAS) measurements were performed at the XAS beamline of the Australian Synchrotron (ANSTO) with monochromatic X-rays (from a Si<111> double crystal monochromator). XAS data was processed and analyzed using Athena and Artemis software [2].

The *in situ* diffuse reflectance infrared Fourier transform (DRIFT) spectra of CO adsorption were acquired with a Bruker Vertex 70 spectrometer equipped with a mercury cadmium telluride (MCT) detector at a resolution of 4 cm^{-1} using 128 scans. All spectra were obtained at room temperature. Prior to CO adsorption, all samples were pretreated with pure argon (Ar) at 300℃ for 30 min. After cooling to room temperature, a 5 vol% CO/Ar mixture was introduced into the cell and the spectra

were collected until the state steady were reached. Subsequently, pure helium was introduced again to remove the gas-phase CO, and the spectra were also collected. Then, all samples were in situ reduced or oxidized at set temperatures with corresponding gases (10 vol% H_2/Ar or pure air) and were purged with pure Ar for 60 min at the respective temperatures. Finally, the spectra of samples were recorded again after absorbing 5 vol% CO/ Ar.

Reaction evaluation

All experiments were conducted in a stirred batch reactor (Parr reactor, Series 4843, 300 mL). In a typical HMF or DFF oxidation experiment, 100 mg of catalyst and 80 mL of HMF or DFF solution (0.03 M in H_2O) were transferred into a high-pressure autoclave reactor charged with 3 MPa pure air. The reactor was then heated to the set temperature with a stirring speed of 700 rpm. The time at which the temperature reached the set point was taken as time zero. The detailed descriptions of reaction conditions can be found in the figure captions and table footnotes. Liquid samples were taken at fixed time intervals and analyzed by high-performance liquid chromatography (HPLC). All substrates and liquid products were analyzed using an Agilent 1290 system equipped with a Bio-Rad Aminex HPX-87H column (300 × 7.8 mm, 9 μm) in conjunction with a Diode array detector (DAD) combined with a refractive index detector (RID). The gaseous products were detected using a Gas Chromatography (GC) equipped with a Thermal Conductivity Detector (TCD) and a Flame Ionization Detector (FID).

Carbon-based concentration (C %) of substrate and major products during the oxidation reactions was calculated according to the following equations:

$$C\,\% = n_{reactants\ or\ products} \times 100 / n_{total}$$

The conversion of reactants, the yield and selectivity of the major products were calculated according to the following equations:

$$The\ conversion\ of\ reactants\ (\%) = (n_0 - n_{reactants}) \times 100) / n_0$$

$$The\ yield\ of\ products\ (\%) = n_{product,i} \times 100 / n_0$$

$$The\ selectivity\ of\ products\ (\%) = n_{product,i} \times 100 / (n_0 - n_{reactants})$$

The carbon molar balance of substrate and all products was calculated according to the following equations:

$$The\ carbon\ balance\ (\%) = n_{total} \times 100) / n_0$$

where n_{total} is the total molar of reactants and products tested on HPLC in the solution. n_0 refers to the initial molar of reactants added before reaction; n_{reactant}, and $n_{\text{product, i}}$ are the molar of reactant and product in the solution after reaction.

The turnover frequency (TOF, h^{-1}) was calculated based on the conversion of HMF/DFF and The Pt dispersion (D):

$$TOF = Moles\ of\ substrate\ consumed/\ (moles\ of\ Pt\ active\ sites*time\ of\ reaction)$$

Here, the active sites were calculated based on the loading Pt concentration tested by XRF and the dispersion of Pt on catalysts obtained using the H_2 titration method introduced in the Characterization part. Here, the consumed substrates (mol) are based on below 30% conversion of HMF in this study, nevertheless, they remain dependable.

Adsorption experiments were carried out in a sealed reactor. A total of 80 mL of a mixed solution containing 0.03 M FFCA and 0.03 M HMF or DFF was prepared and transferred, along with 100 mg of catalyst, into reactors of identical volume. The reactors were sealed and stirred at room temperature overnight. The concentration of

FFCA both in the initial solution and after stirring was measured to evaluate the adsorption of FFCA on the catalysts. The absorption rate (%) was calculated based on the following equations:

$$\textit{The absorption rate (\%)} = (n_0 - n_{tirring}) \times 100) / n_0$$

where n_0 refers to the initial molar of FFCA tested on HPLC in the mixed solution; n_{stirring} is the molar of FFCA in the solution after stirring.

<u>Density functional theory calculation (DFT)</u>

All calculations were performed using the density functional theory (DFT) technique using the Vienna ab initio simulation package (VASP). Spin-polarized calculations were performed using the generalized gradient approximation (GGA) combined with the Perdew–Burke–Ernzerhof (PBE) method to describe the exchange and correlation energy in all the calculations [3]. The density functional dispersion correction (DFT-D3) method was used to simulate van der Waals interactions during the DFT calculation. The projector-augmented wave (PAW) method was used to describe the pseudopotential. Titanium 3d, 4s; oxygen 2p, 2s; Bismuth 4d 5s; and platinum 4d, 5s electrons were considered valence electrons, and an energy cutoff of 400 eV was used for basis-set expansion. For geometry optimization calculations, forces were converged below 0.03 eV/Å. The SCF convergence energy was 1×10^{-4} Ha. A $1 \times 1 \times 1$ k-point mesh was used to perform all the calculations. During the calculation, we used +U for correction, all the values were 3.5 eV for Ti.

Three-layer slab model surfaces of TiO_2 (101) substitution was built to calculate the adsorption energies and Gibbs free energies for the calculation of oxygen vacancy formation energy. Two *p* (3 × 3) unit cell expansions were used to simulate the Bi/TiO_2, Pt/TiO_2 and $Pt\text{-}Bi/TiO_2$ surfaces, with a vacuum of 15 Å applied under

periodic boundary conditions. The energy of oxygen vacancy formation, E_v, for an oxygen atom released from TiO_2 on the surface to generate an oxygen vacancy is defined as follows:

$$E_v = E_{defect} + \mu_O - E_{bulk}$$

where E_{bulk} is the total clean relaxation energy of the surface slab, μ_0 is the chemical potential of oxygen, and E_{defect} is the total energy of the slab with an oxygen atom removed from the surface. Because the calculations are performed at 0 K and fixed cell volume, the differences in Gibbs free energy should be equal to the differences in total energy. Thus, the lower the Ev is, the more easily oxygen ions are released, which promotes the migration of oxygen atoms

The binding/adsorption energy (E_{ads}) of the HMF, DFF, and the other molecules on the surface was calculated as follows:

$$E_{ads} = E_{adsorbate+surface} - E_{surface} - E_{gas}$$

where $E_{surface}$ refers to the clean surface relaxation energy of the surface slab, E_{gas} denotes the energy of the isolated gas-phase molecule under vacuum conditions and $E_{adsorbate+surface}$ represents the total energy of the adsorbate-surface system.

According to the definition of ΔG [4,5],

$$\Delta G = \Delta E + \Delta E_{ZPE} - T\Delta S + \Delta G_U + \Delta G_{pH} + \Delta G_{field}$$

ΔE was directly calculated by DFT. ΔE_{ZPE} was obtained by calculating the vibration frequencies of the intermediates, accounting for the adsorbate vibrations at 0 K. Given the conservation of elements before and after each calculation step, ΔE_{ZPE} can be approximated as zero. Since the temperature is defined as 0 K, TΔS is also zero [6]. ΔG_{pH} is the contribution of H^+ concentration change to the Gibbs free energy. Since the absence of pH changes in the gas-solid reaction, it can be ignored as zero [7]. The

contribution from the electrode potential (ΔG_U) is irrelevant, as electrocatalysis is not involved in this study. Finally, the correction term for electric double layer free energy (ΔG_{field}) has negligible effect on overall free energy and thus also ignored [5].

The barrier energy, ΔE, which represents the disparity between transition state energy difference and standard product formation enthalpy, is defined as follows. For example, the energy barrier calculation of *-CH_2OH to *-CHO and OH on Pt/TiO_2 is exemplified in this study:

$$\Delta E = (E_{CH2OH\text{-}Pt/TiO2} - E_{stab\text{-}Pt/TiO2}) + 2 \times (E_{O\text{-}Pt/TiO2} - E_{stab\text{-}Pt/TiO2}) - (E_{CHO\text{-}Pt/TiO2} - E_{stab\text{-}Pt/TiO2}) + 2 \times (E_{OH\text{-}Pt/TiO2} - E_{stab\text{-}Pt/TiO2})$$

where $E_{\mathrm{CH2OH\text{-}Pt/TiO2}}$, $E_{\mathrm{O\text{-}Pt/TiO2}}$, $E_{\mathrm{CHO\text{-}Pt/TiO2}}$ and $E_{\mathrm{OH\text{-}Pt/TiO2}}$ are the energies of the *-CH_2OH, O, *-CHO and OH adsorbed on Pt/TiO_2, $E_{\mathrm{stab\text{-}Pt/TiO2}}$ is the energy of the Pt/TiO_2 surface slab. As the calculations are performed at 0 K at a fixed cell volume, the differences in the Gibbs free energy should equal the differences in the total energy. By this definition, the negative value of E_{ads} corresponds to exothermic and spontaneous adsorption processes.

SUPPLEMENTARY FIGURES AND SCHEMES

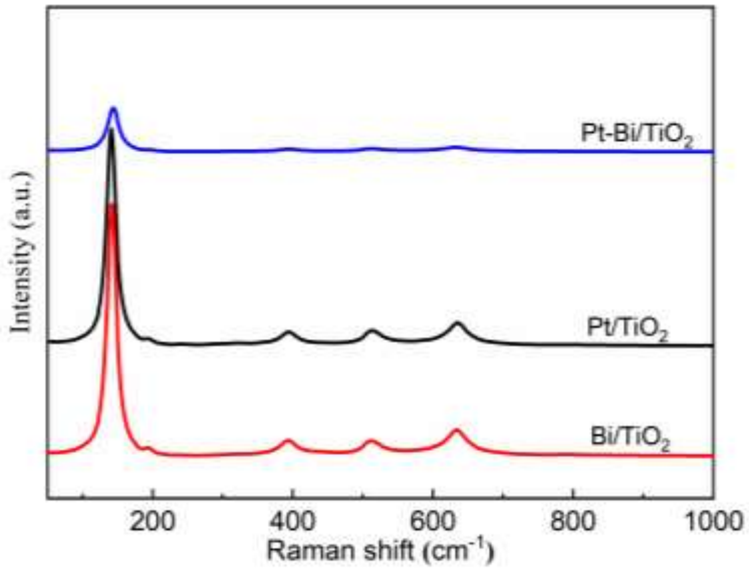

Figure S1 Raman spectra of TiO_2-based catalysts.

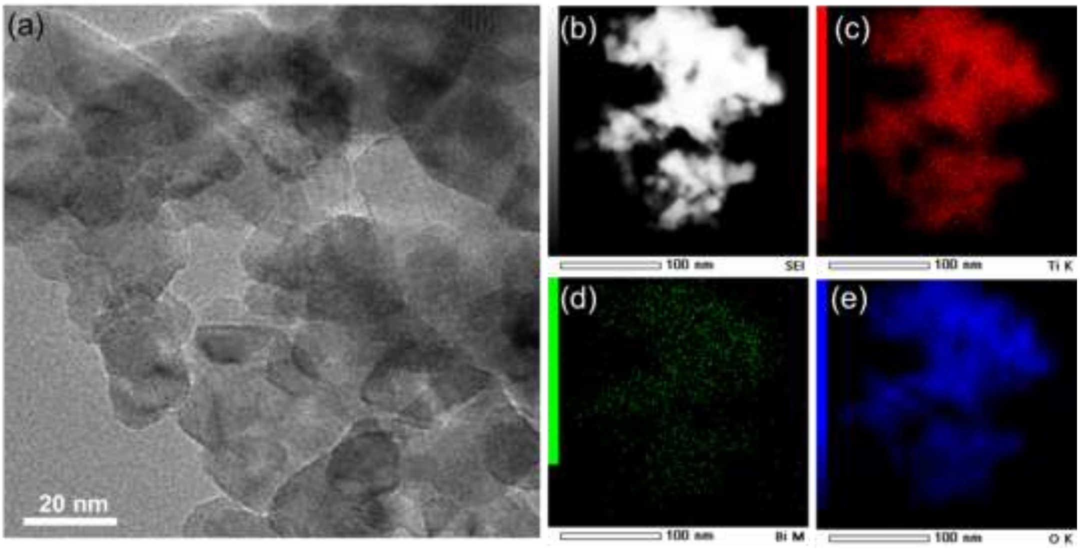

Figure S2 HRTEM (a-b) and element mapping (c-e) images of Bi/TiO_2.

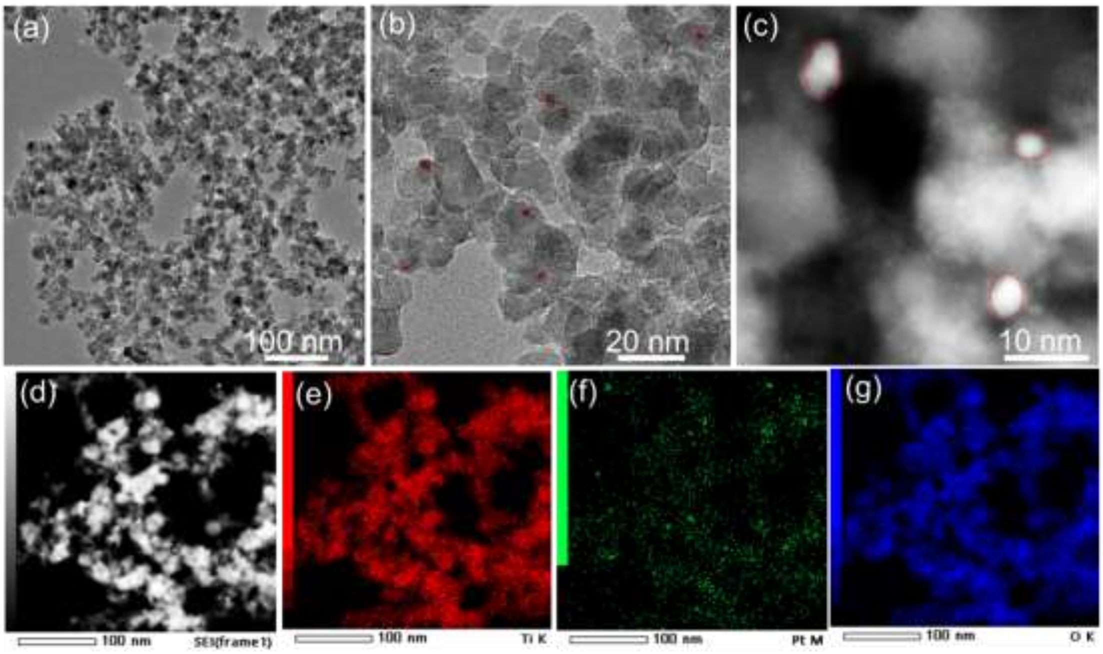

Figure S3 (a-b) HRTEM, (c-d) HAADF-STEM and (e-g) element mapping images of Pt/TiO_2.

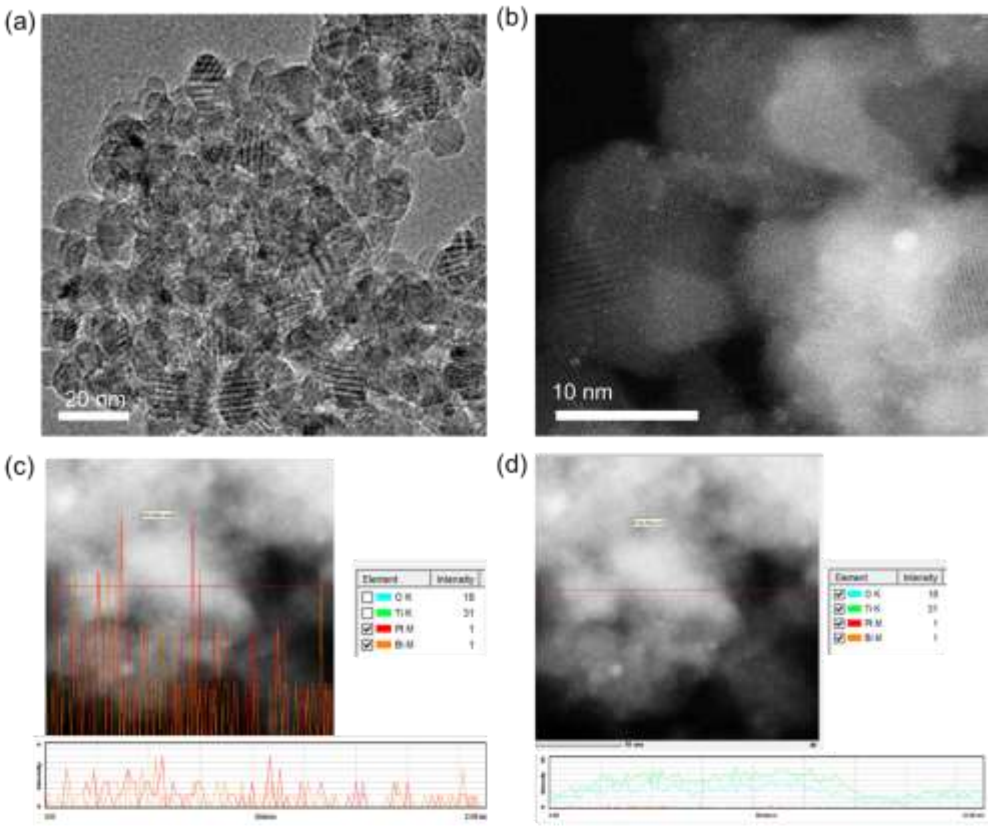

Figure S4 (a) HRTEM, (b) HAADF-STEM and (c-d) linear scan images of Pt-Bi/TiO_2.

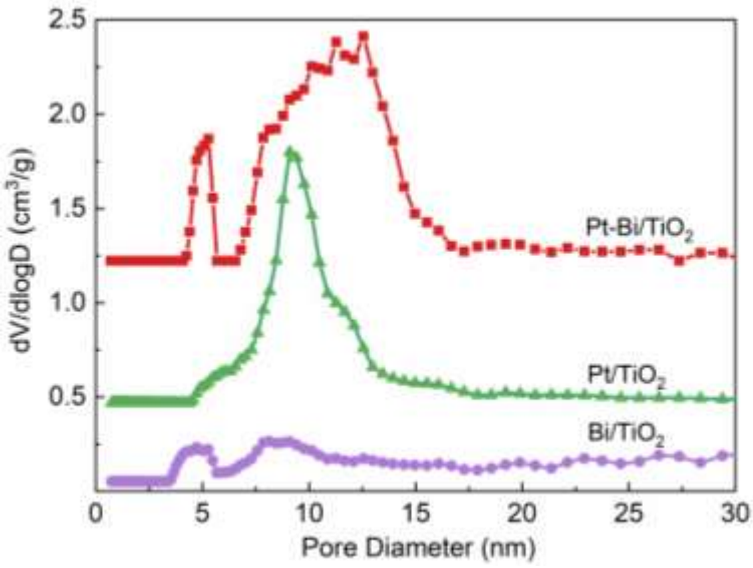

Figure S5 The pore size distribution curves of different TiO_2-based samples. The pore size distribution curves were determined using DFT method.

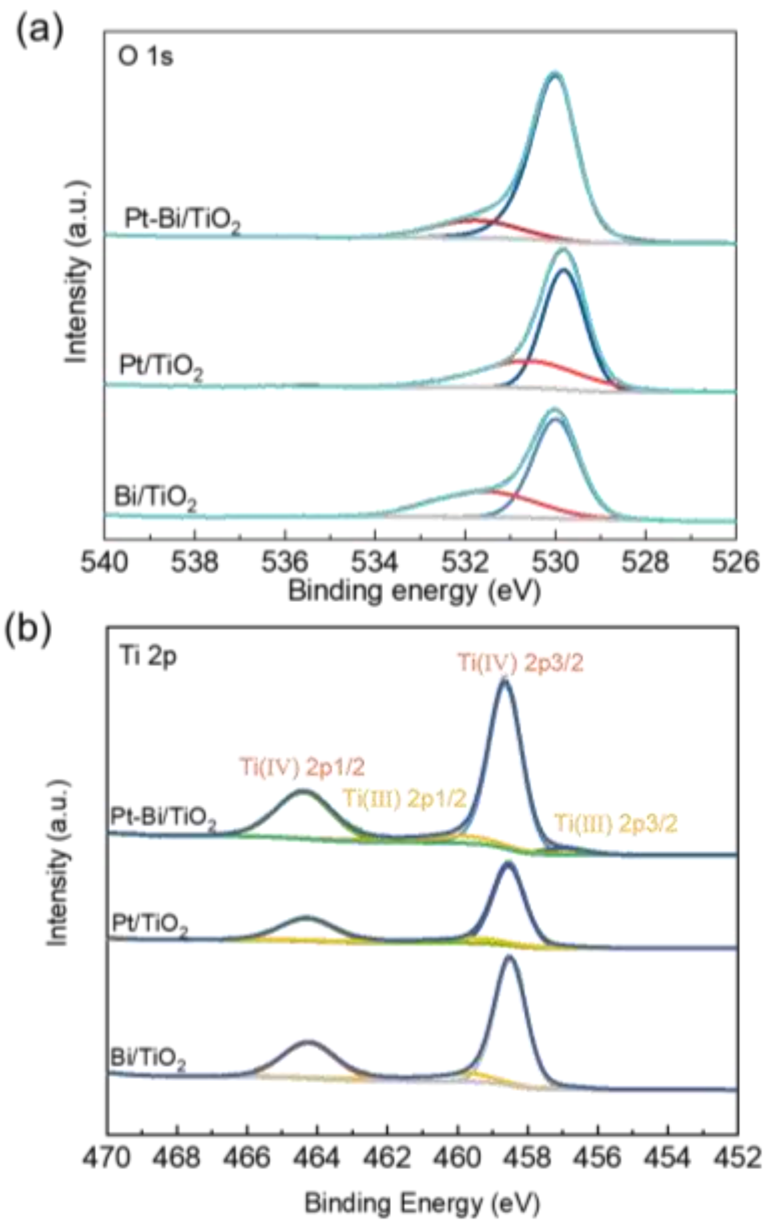

Figure S6 XPS of TiO_2-based samples, (a) O 1s and (b) Ti 2p spectra.

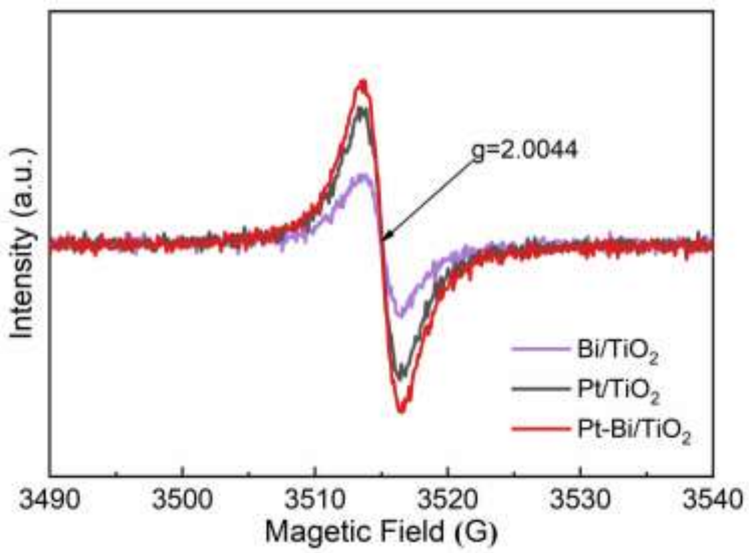

Figure S7 EPR spectra of TiO_2-based samples.

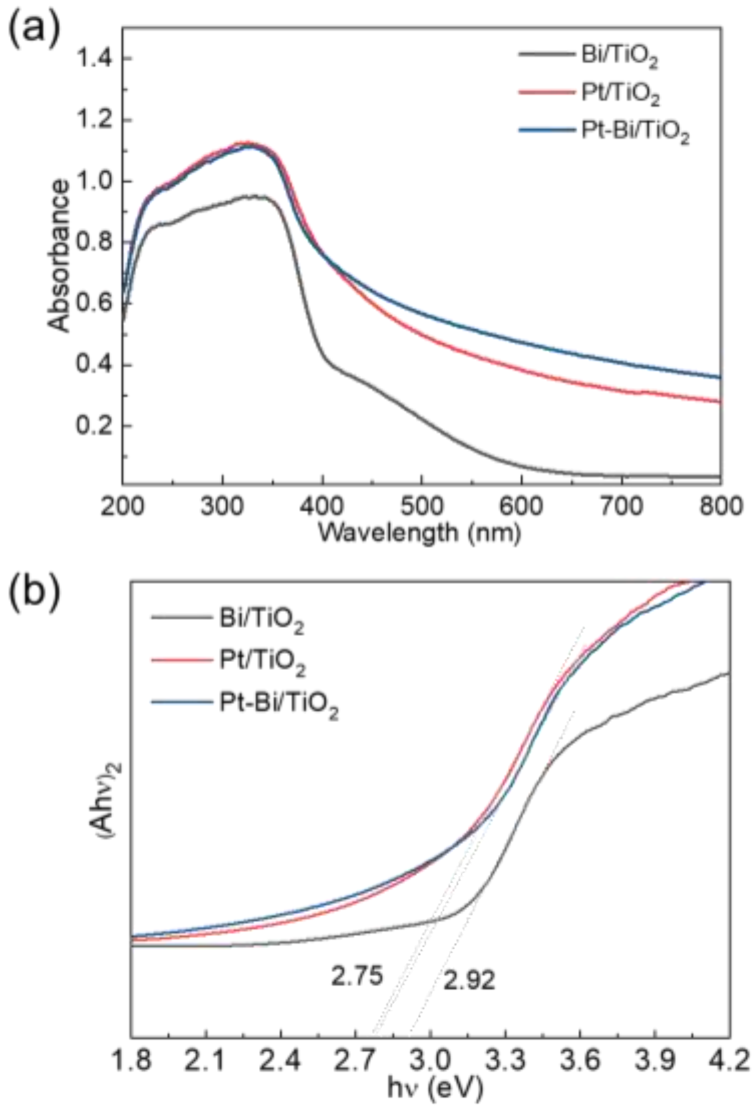

Figure S8 UV/Vis DRS of TiO_2-based samples.

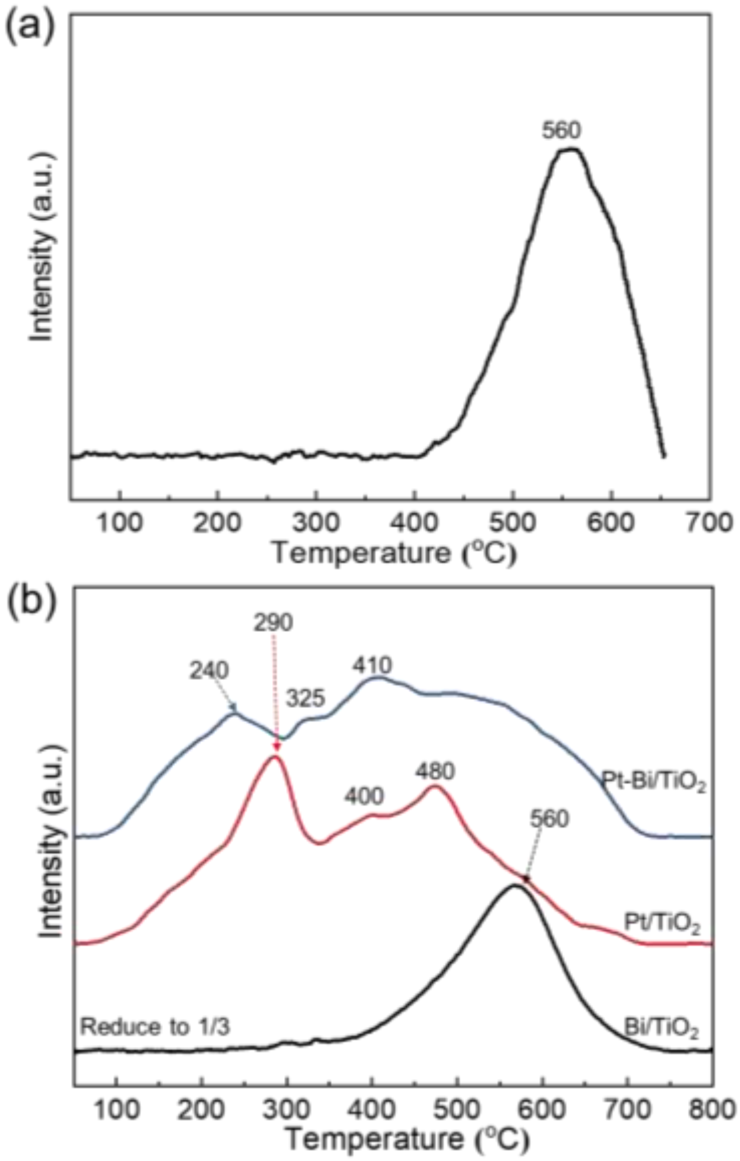

Figure S9 H_2-TPR profile of TiO_2-based materials.

The reducibility of these catalysts was examined by H_2-TPR. TiO_2 (Figure S9a) and Bi/TiO_2 (Figure S9b) showed the same reduction peak at 560℃ ascribed to the reduction of Ti(Ⅳ) to Ti(Ⅲ) species. The reduction peak of Ti(Ⅳ) shifted to lower temperature (*ca.* 480℃) when Pt incorporated into TiO_2. This phenomenon demonstrated that the presence of Pt in the TiO_2 catalyst can promote the reduction of Ti(Ⅳ) which could be due to the spillover of active H atoms on Pt species to Ti species, and then Ti(Ⅳ) would be easily reduced to Ti(Ⅲ). And the peaks centered at ca. 250 and 400℃ on Pt/TiO_2 could be attributed to the reduction of cationic $Pt^{\delta+}$

nanoparticles and/or $Pt^{\delta+}$ single atoms to metallic Pt(0) species, respectively. Further adding Bi into Pt/TiO_2, Ti(Ⅳ) reduction peak further shifted slightly to lower temperature centered at *ca.* 410℃. Meanwhile the ones due to $Pt^{\delta+}$ species also shifted to lower temperatures. This indicates Bi doping enhances the electron mobility on material, boosting the electron transfer from H_2 to $Ti^{\delta+}$ and/or $Pt^{\delta+}$ and further lowering the reduction temperature.

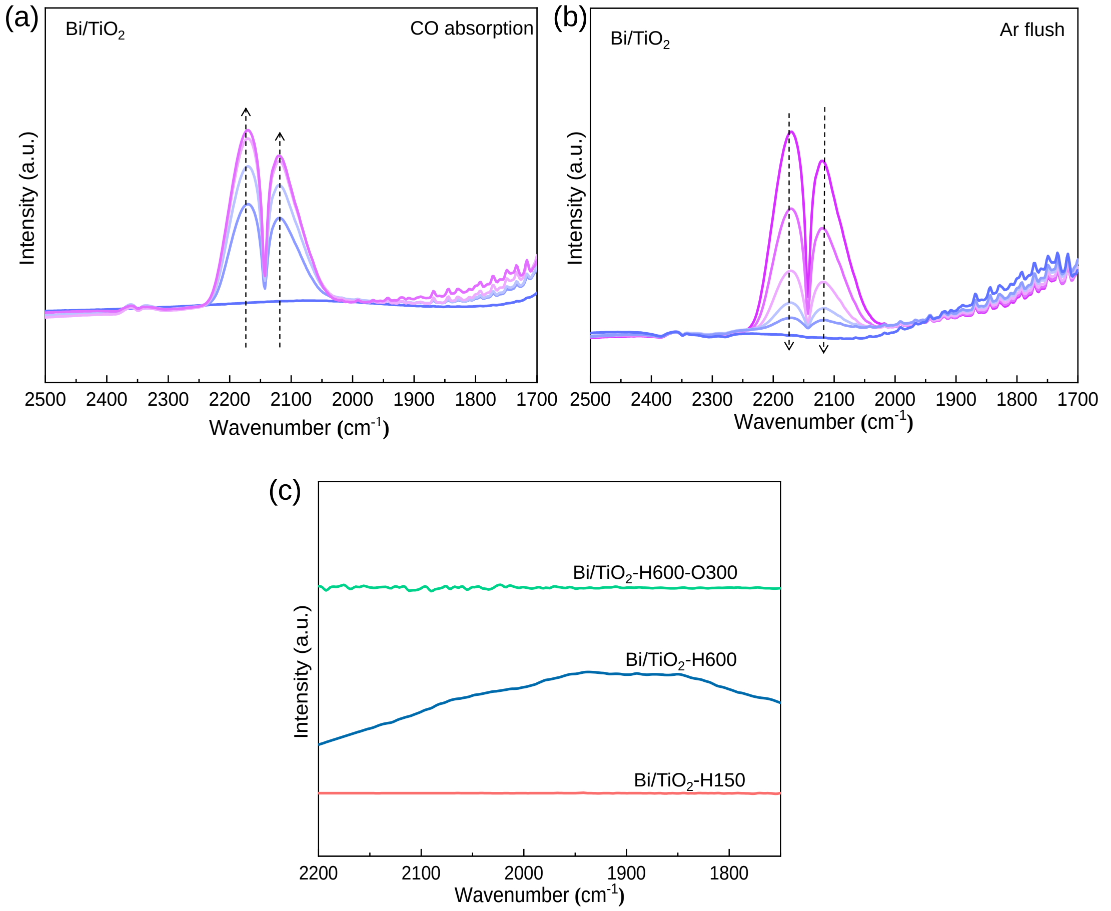

Figure S10 (a-b) *In situ* DRIFT spectra of CO adsorption at room temperature on Bi/TiO_2, saturation coverage during Ar flushing and (c) FTIR spectra of CO adsorbed on Bi/TiO_2-Hx and Bi/TiO_2-Hx-Oy after Ar purging.

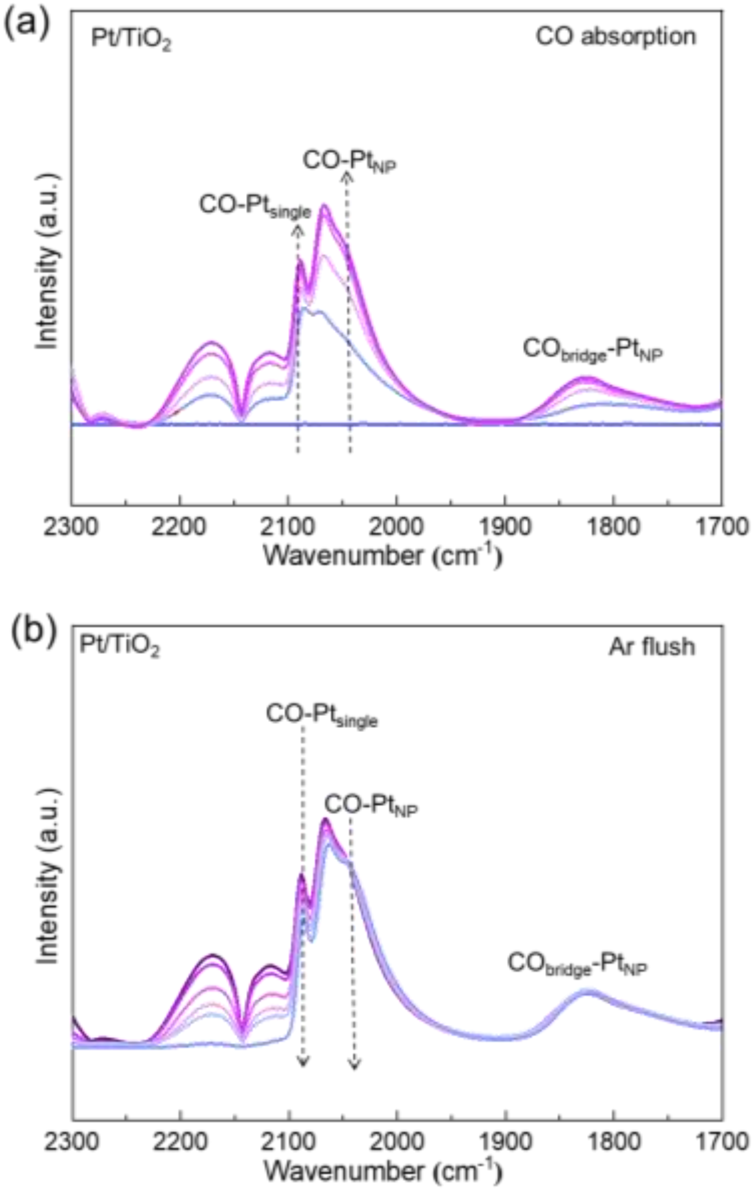

Figure S11 (a) *In situ* DRIFT spectra of CO adsorption at room temperature on Pt/TiO_2 and (b) saturation coverage during Ar flush.

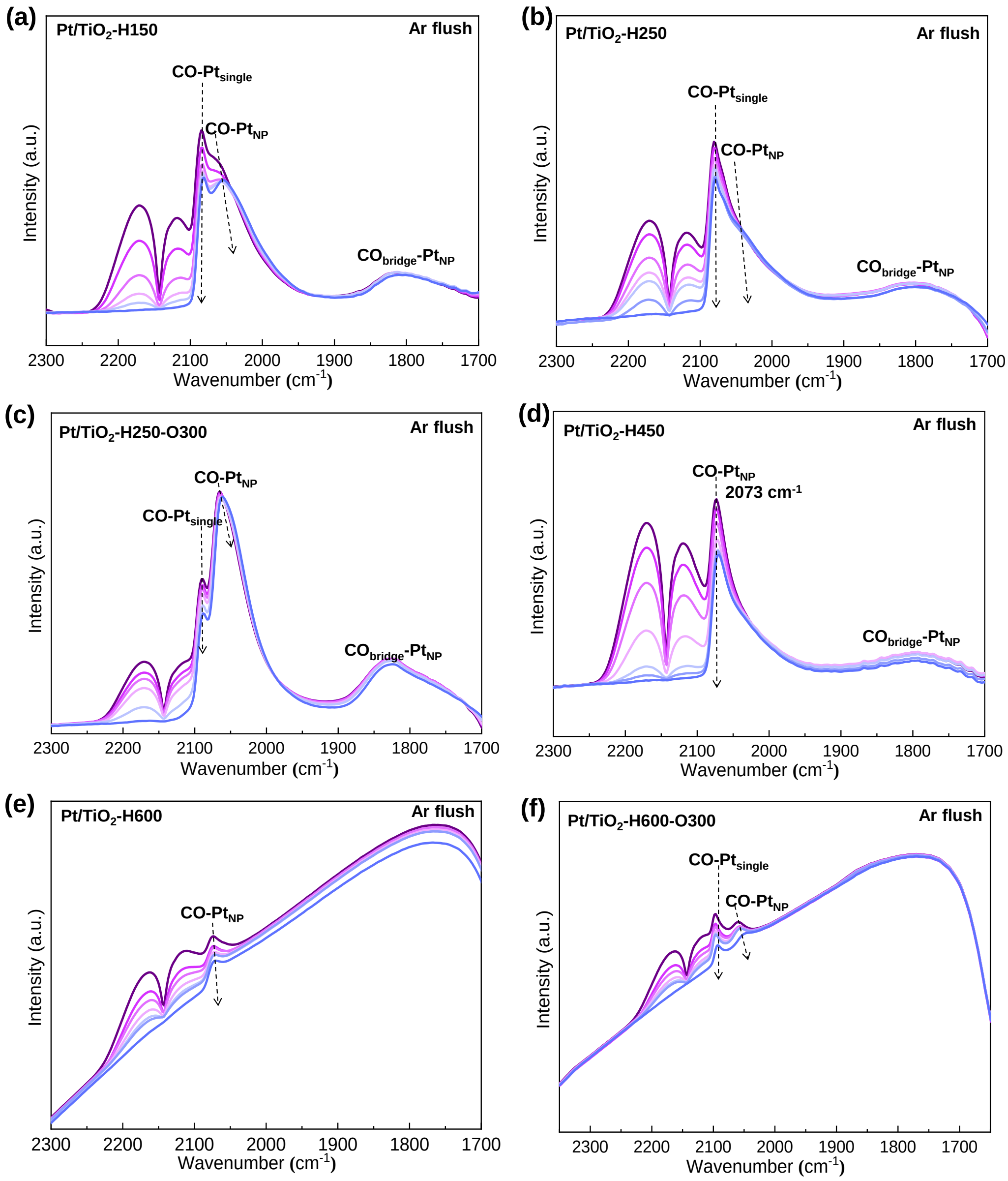

Figure S12 *In situ* DRIFT spectra of Pt/TiO_2 with different treatment methods showing CO adsorption after Ar purging at room temperature.

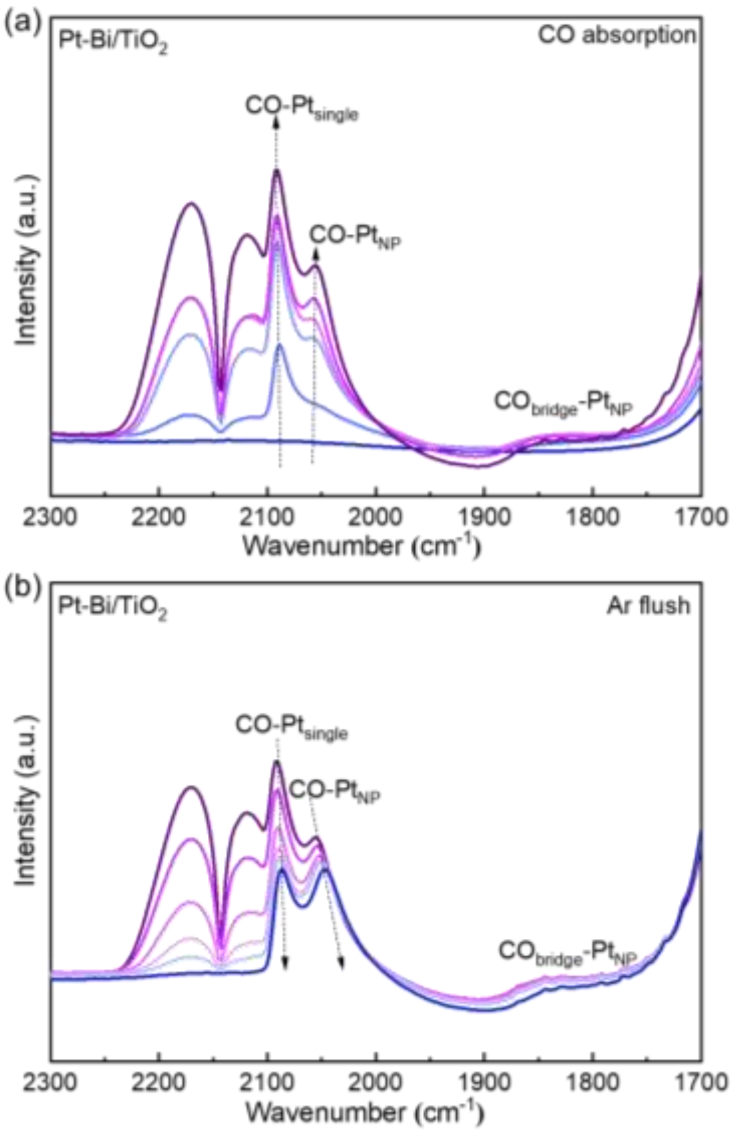

Figure S13 (a) *In situ* DRIFT spectra of CO adsorption at room temperature on Pt-Bi/TiO_2 and (b) saturation coverage during Ar flush.

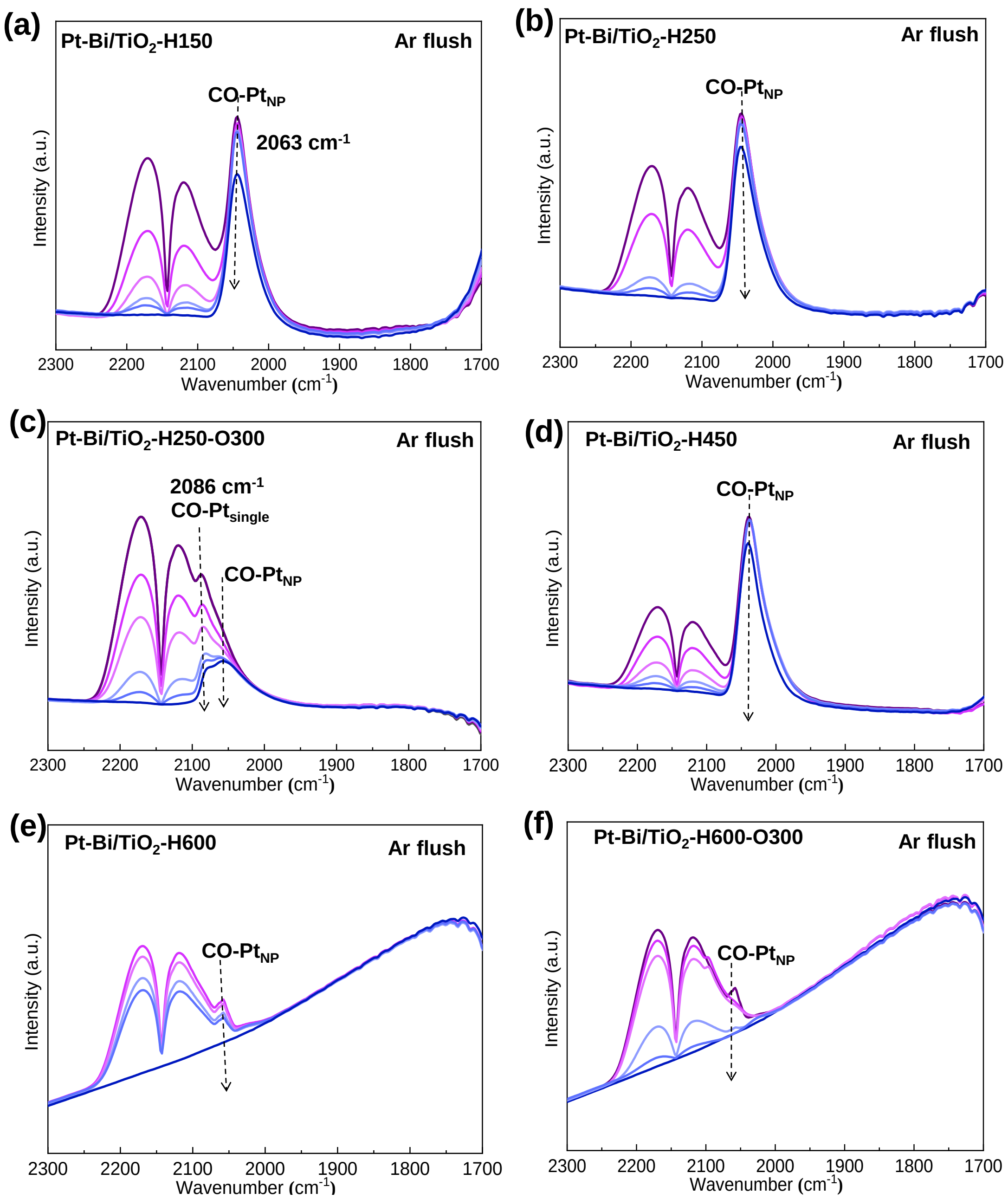

Figure S14 *In situ* DRIFT spectra of Pt-Bi/TiO_2 with different treatment methods showing CO adsorption after Ar purging at room temperature.

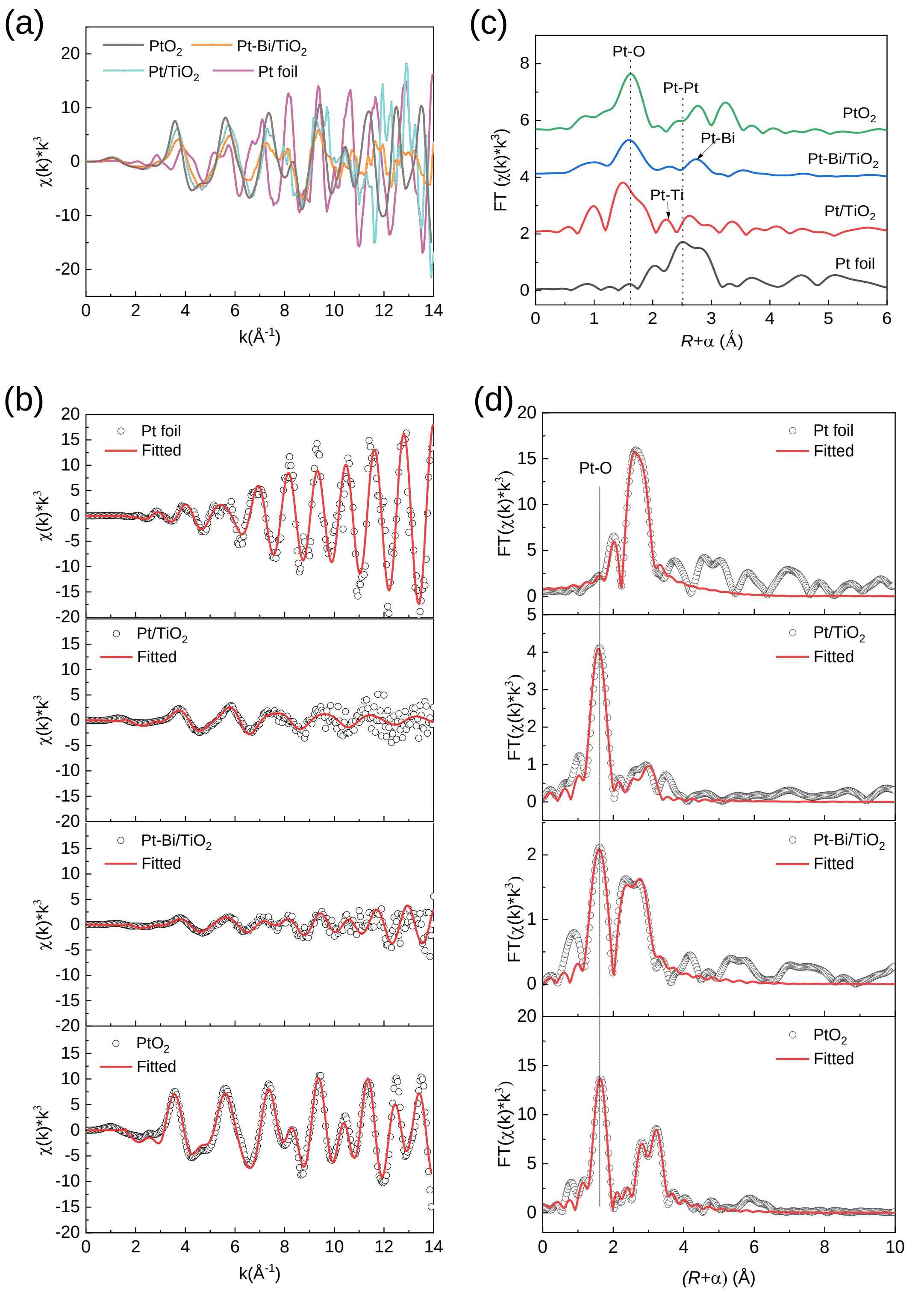

Figure S15 EXAFS data (points) and the curve-fit (line) in (a-b) K space and (c-d) R space (d is FT magnitude) shown in k3 weighted of Pt/TiO_2, and Pt-Bi/TiO_2. The data are k3 weighted and not phase corrected. For comparison, the EXAFS spectra of standards samples (Pt foil and PtO_2) are also included.

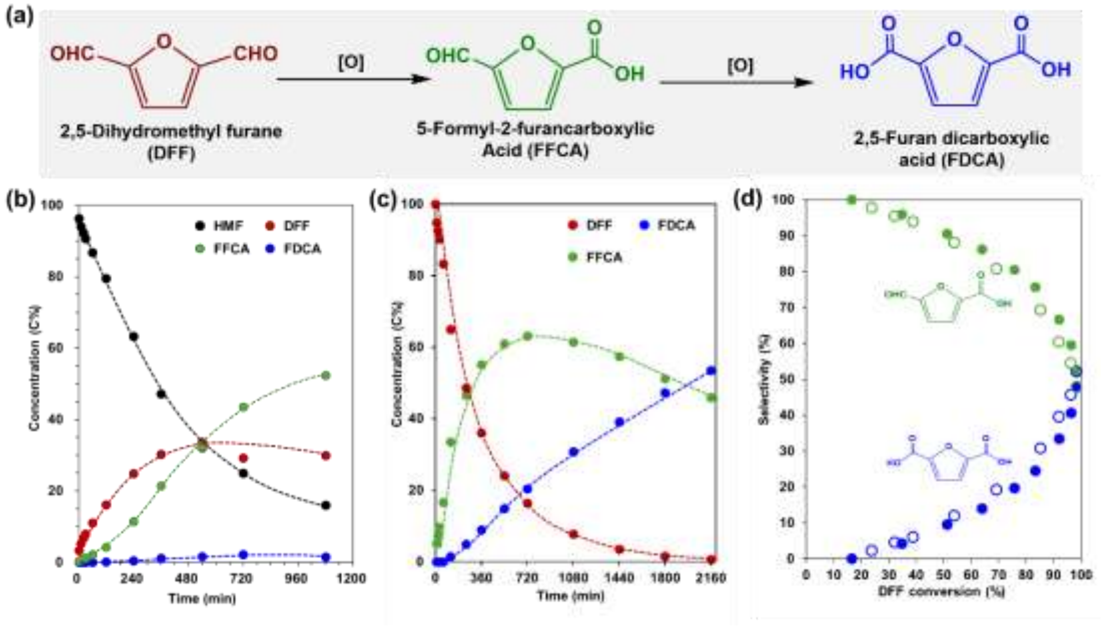

Figure S16 (a) The scheme of DFF oxidation process and carbon-based concentration-time profiles of (b) HMF and (c) DFF oxidation on Pt/TiO_2 in water, and (d) the main products selectivity as function of DFF conversion over Pt/TiO_2 and Pt-Bi/TiO_2. Solid symbol in d is for Pt/TiO_2 and hollow symbol for Pt-Bi/TiO_2; Reaction conditions: 100 mg catalyst, 80 ml 0.03M DFF aqueous solution, 3 MPa (ambient temperature) Air, stirred at 700 r.p.m., 150℃.

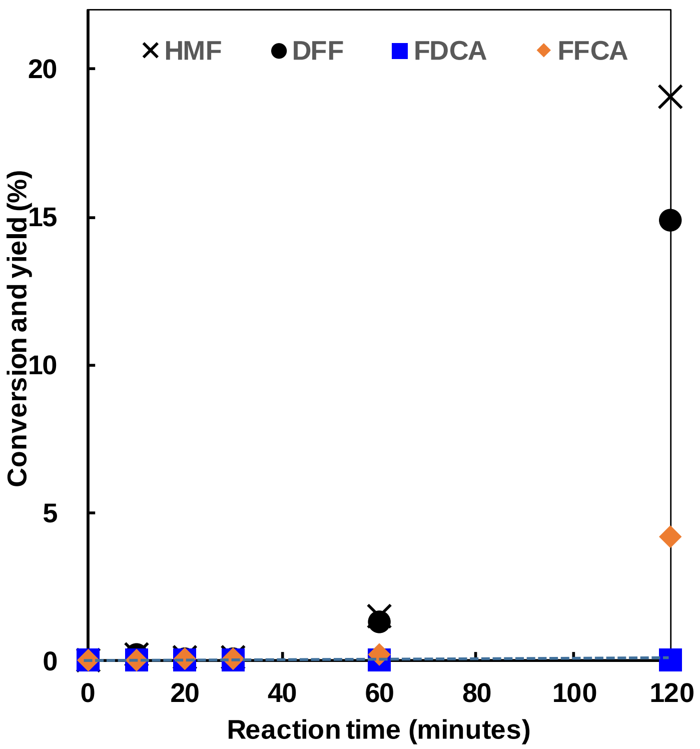

Figure S17 Time dependence of HMF conversion as well as the yield towards the major products over Bi/TiO_2 catalysts. Reaction conditions: 100 mg catalyst, 80 ml 0.03 M HMF aqueous solution, 3 MPa (ambient temperature) Air, stirred at 700 r.p.m., 150℃.

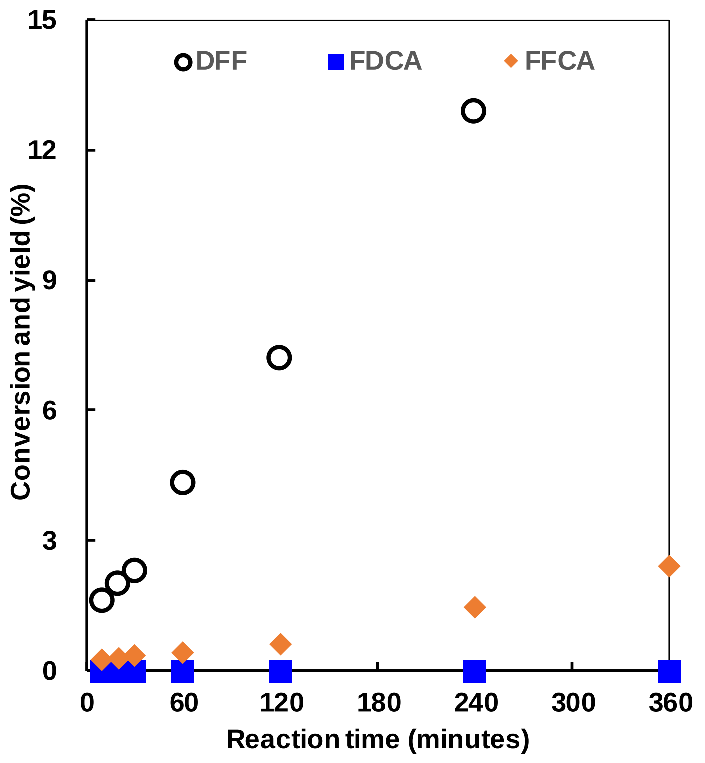

Figure S18 Time dependence of DFF conversion as well as the yield towards the major products over Bi/TiO_2 catalysts. Reaction conditions: 80 mL of DFF water solution (0.03 mol/L); 100 mg catalyst; 150℃, 3MPa air, 700 rpm.

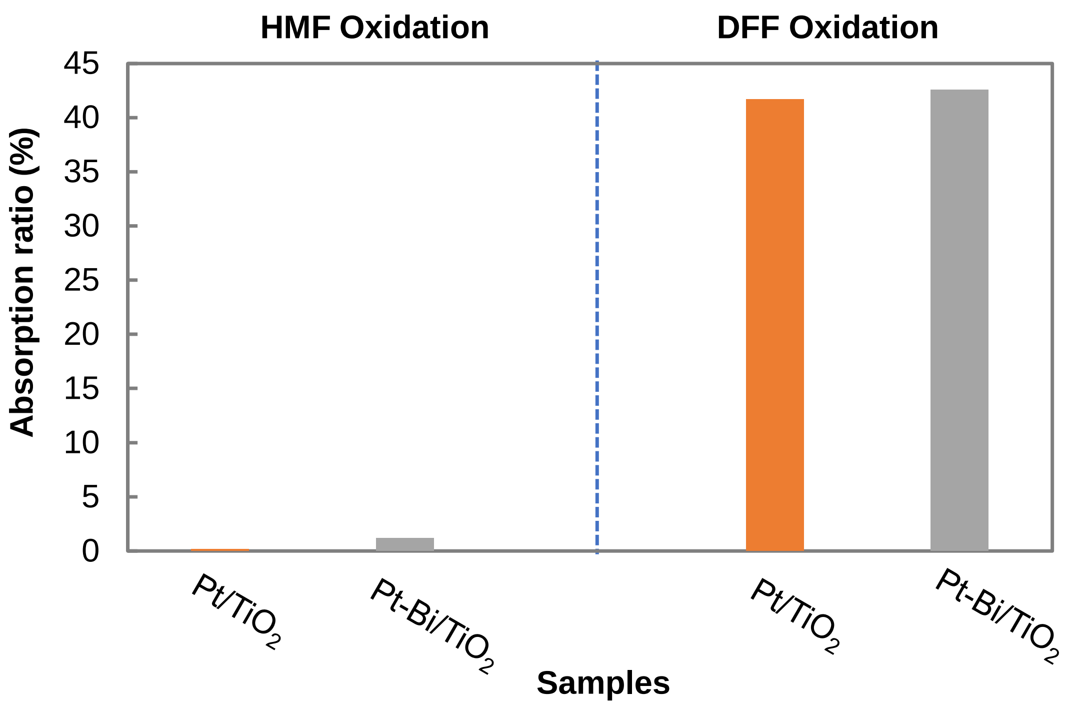

Figure S19 The absorption ratio of FFCA over Pt/TiO_2 and Pt-Bi/TiO_2 in the presence of HMF or DFF. The absorption condition: ambient temperature for overnight, 0.03M FFCA mixed with 0.03 M HMF or DFF, 80 ml solution, 100 mg catalyst and 700 r.p.m.

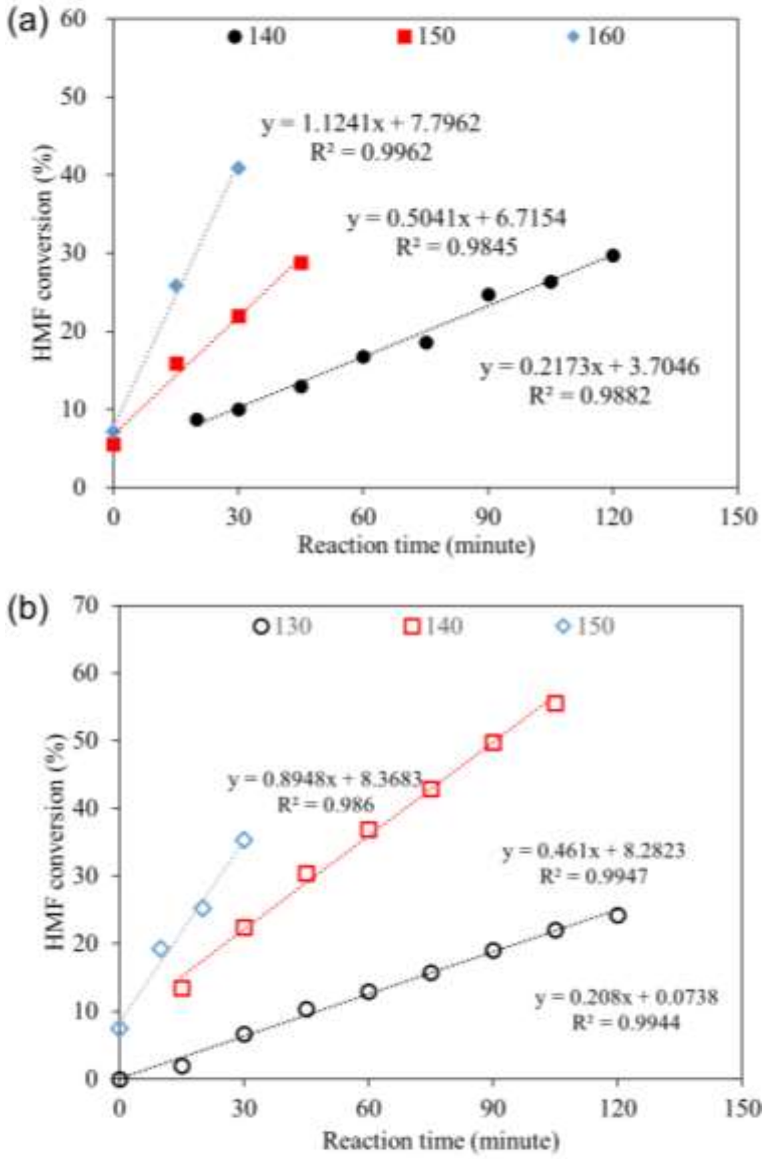

Figure S20 The kinetics profiles of HMF oxidation (fitted by first-order assumption) with (a) Pt/TiO_2 and (b) Pt-Bi/TiO_2.

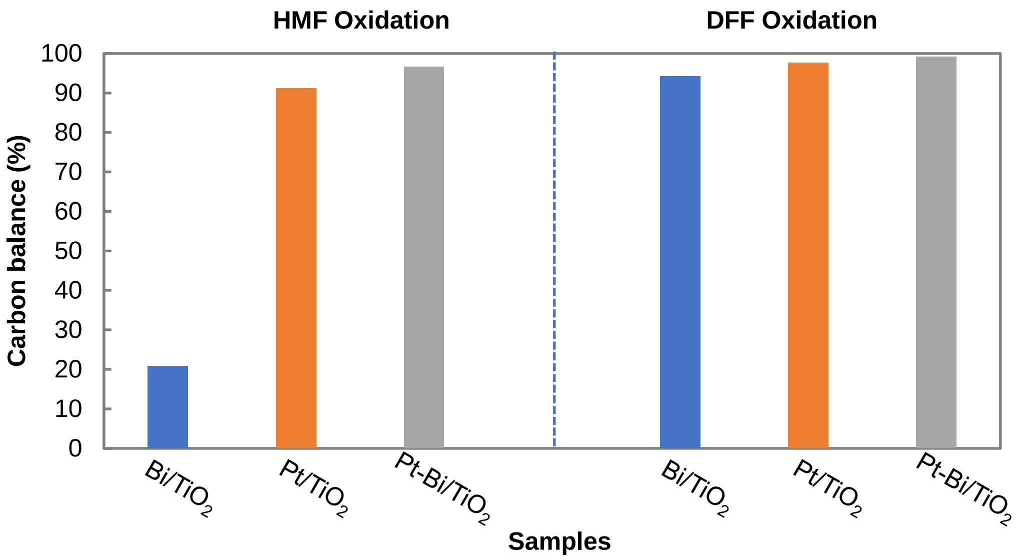

Figure S21 The carbon molar balance of the HMF and DFF oxidation reaction over Bi/TiO_2, Pt/TiO_2, and Pt-Bi/TiO_2, respectively. The carbon balance was calculated by the total molar of all detected products and unreacted substrate in the reaction solution divided by the original molar of substrate, where the gaseous products were not contained.

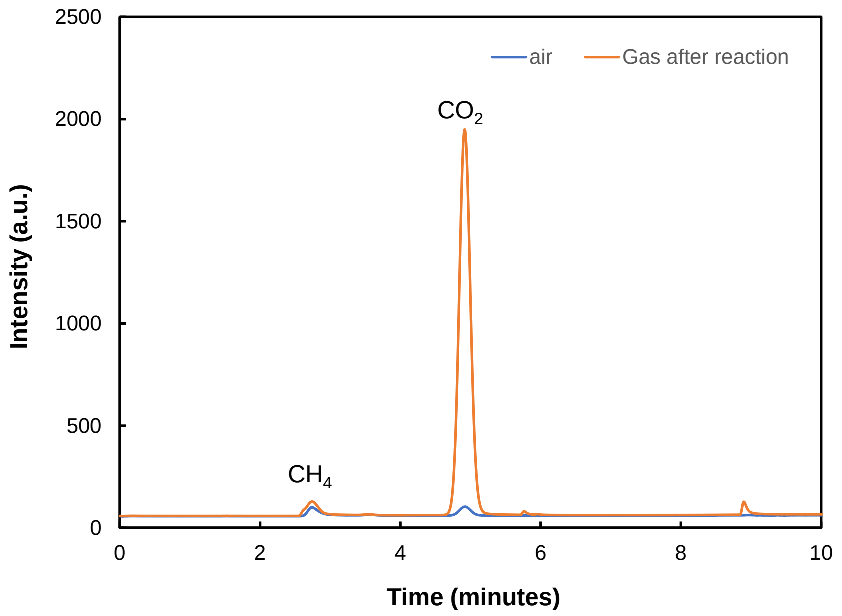

Figure S22 The GC analysis of the gas collected after the reaction of HMF oxidation over Pt-Bi/TiO_2, relative to the air baseline.

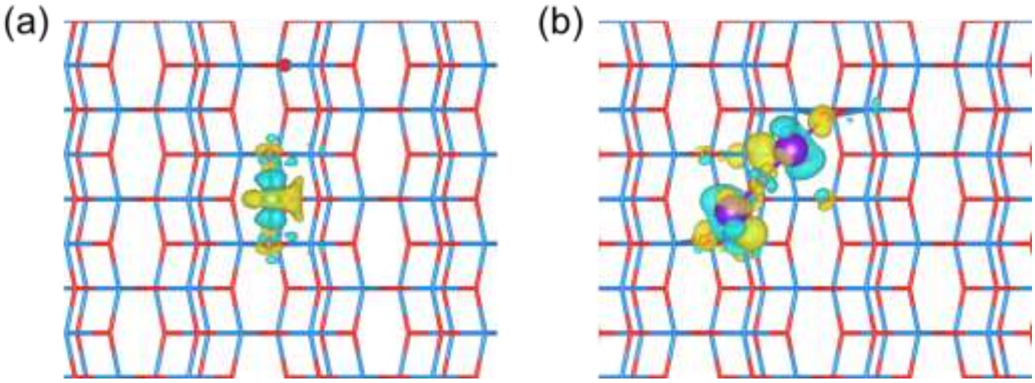

Figure S23 Charge density difference plots on the surface of (a) Pt/TiO_2 and (b) Bi/TiO_2. Yellow and blue respectively represent an increase and decrease in electron cloud density, with an interface value of 0.005 e ƀohr^{-3}.

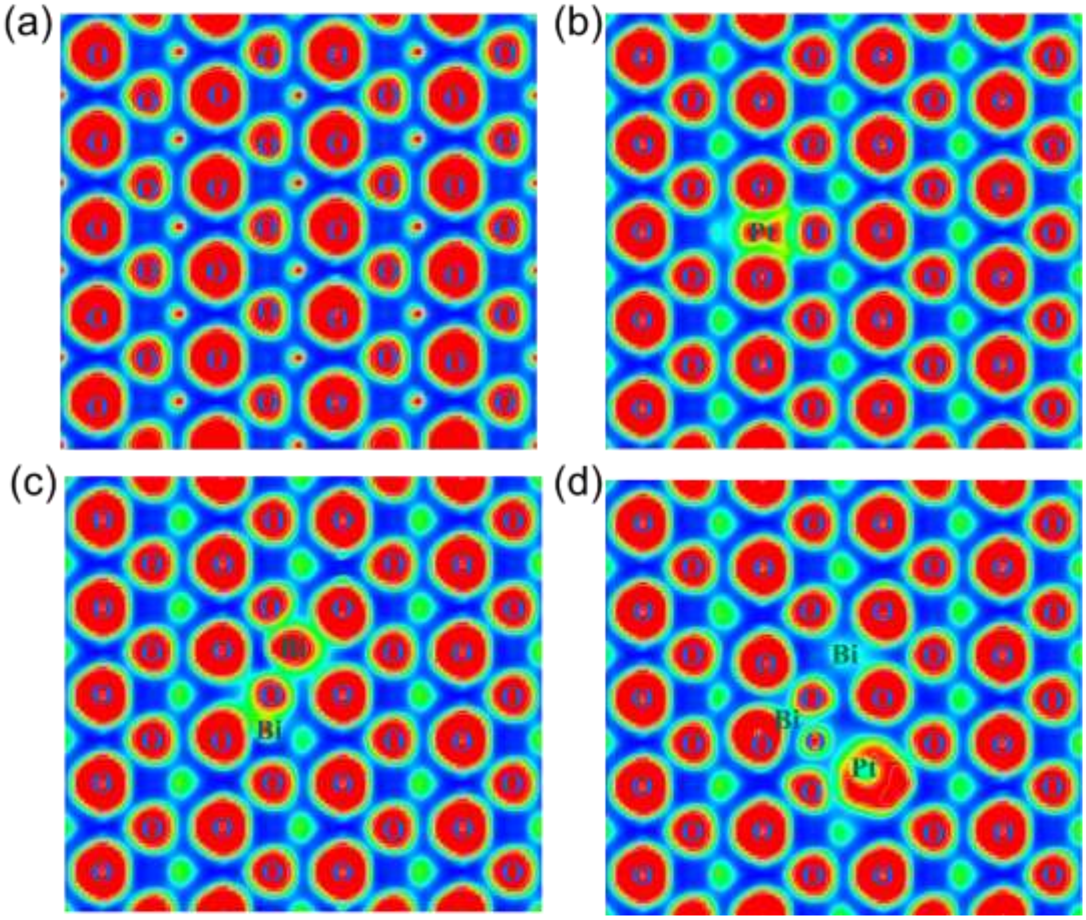

Figure S24 Electron locational Function (ELF) plot on the surface of (a) TiO_2，(b) Pt/TiO_2, (c) Bi/TiO_2 and (d) Pt-Bi/TiO_2.

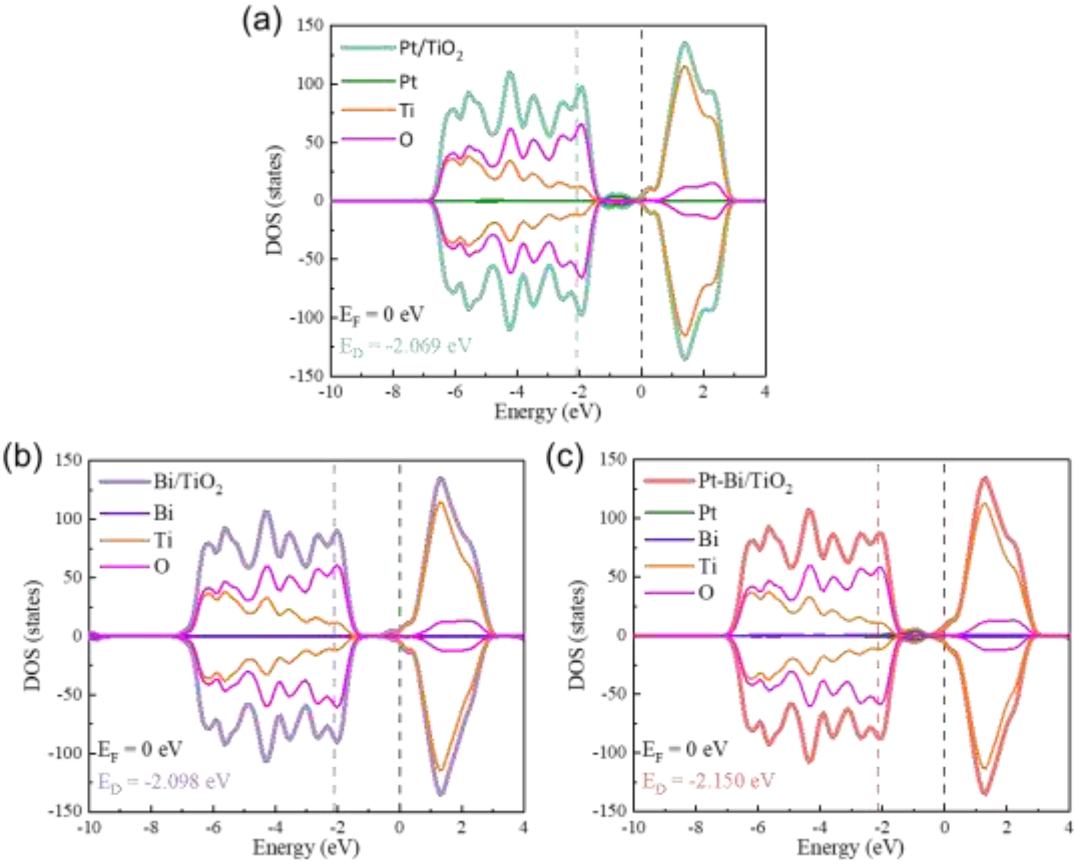

Figure S25 Density of States (DOS) of (a) Pt/TiO_2, (b) Bi/TiO_2 and (c) Pt-Bi/TiO_2.

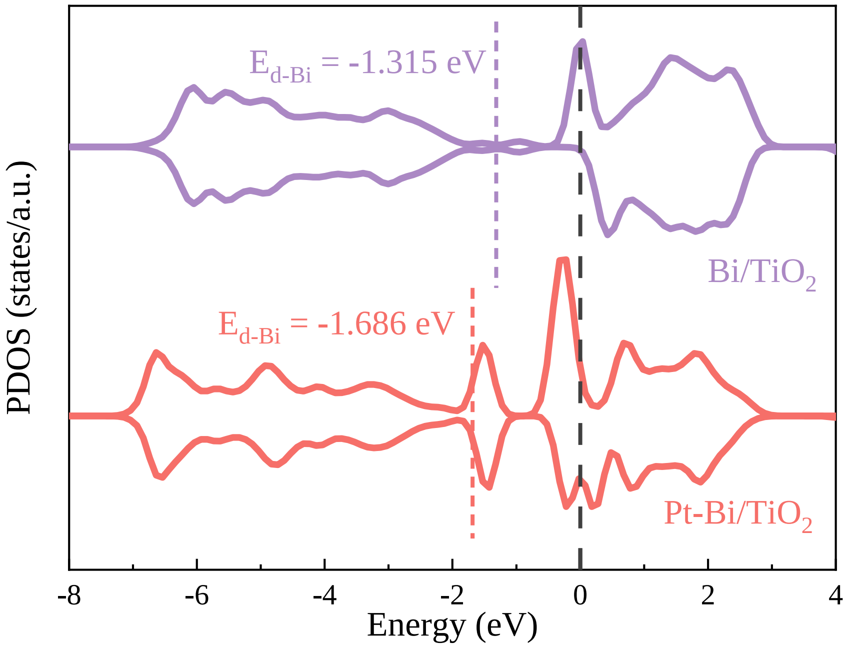

Figure S26 Project Density of States (PDOS) of Bi/TiO_2 and $Pt-Bi/TiO_2$.

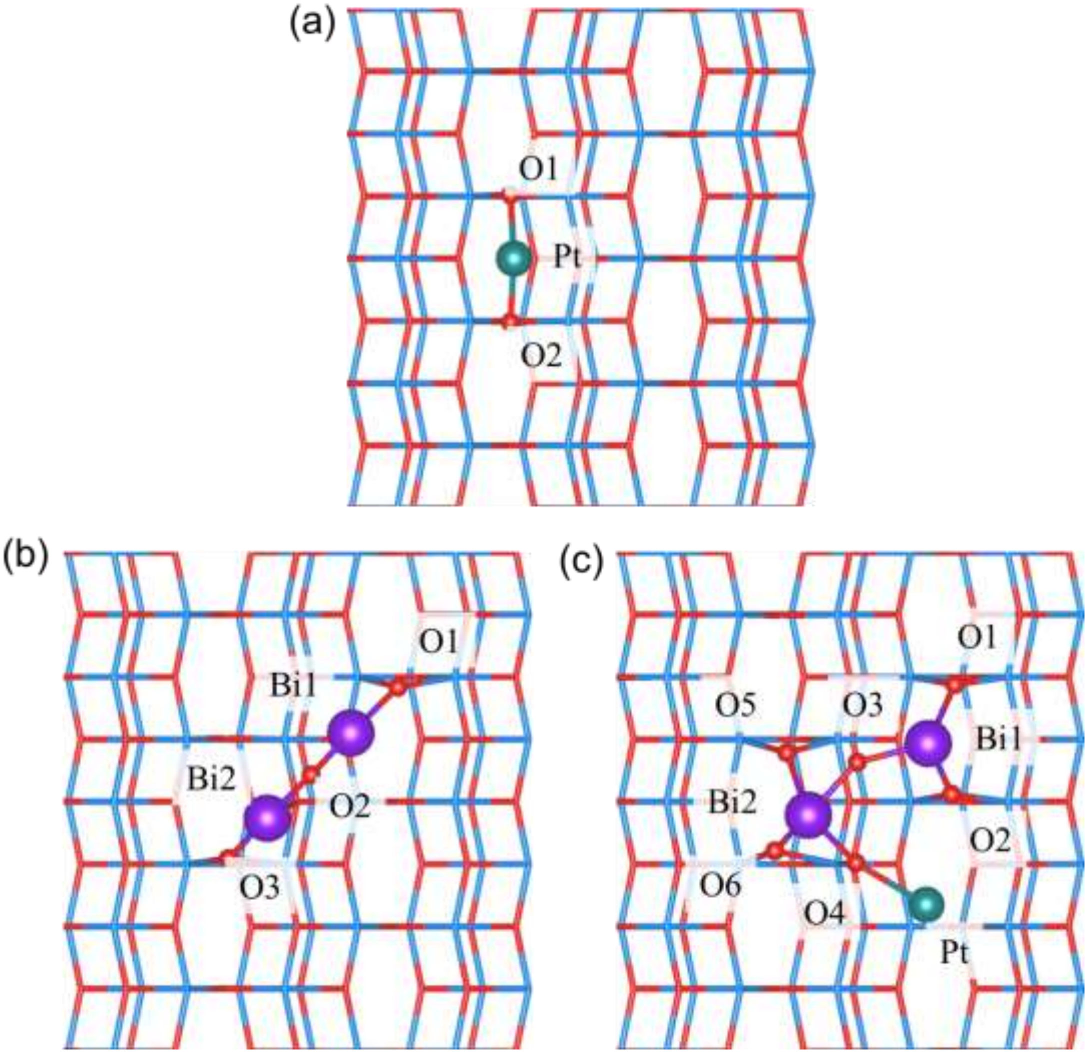

Figure S27 Model of (a) Pt/TiO_2, (b) Bi/TiO_2 and (c) Pt-Bi/TiO_2 in Bader charge calculation.

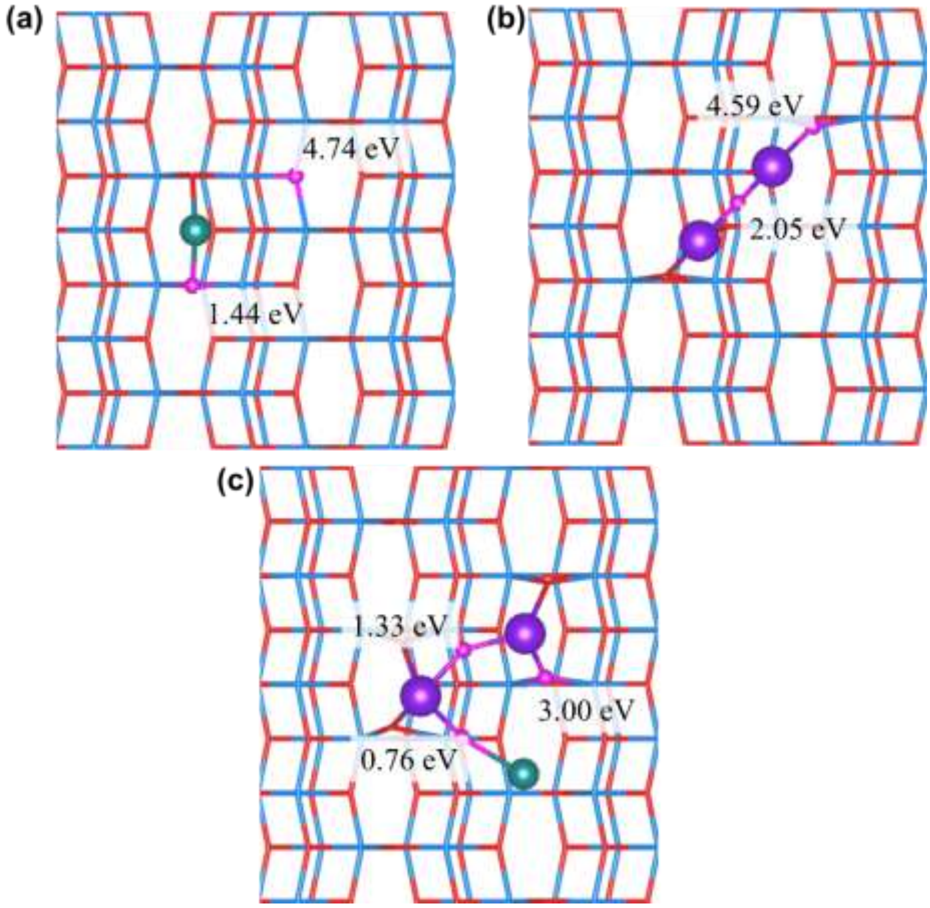

Figure S28 The plot of oxygen vacancies (OVs) positions and their formation energies on (a) Pt/TiO_2, (b) Bi/TiO_2 and (c) Pt-Bi/TiO_2.

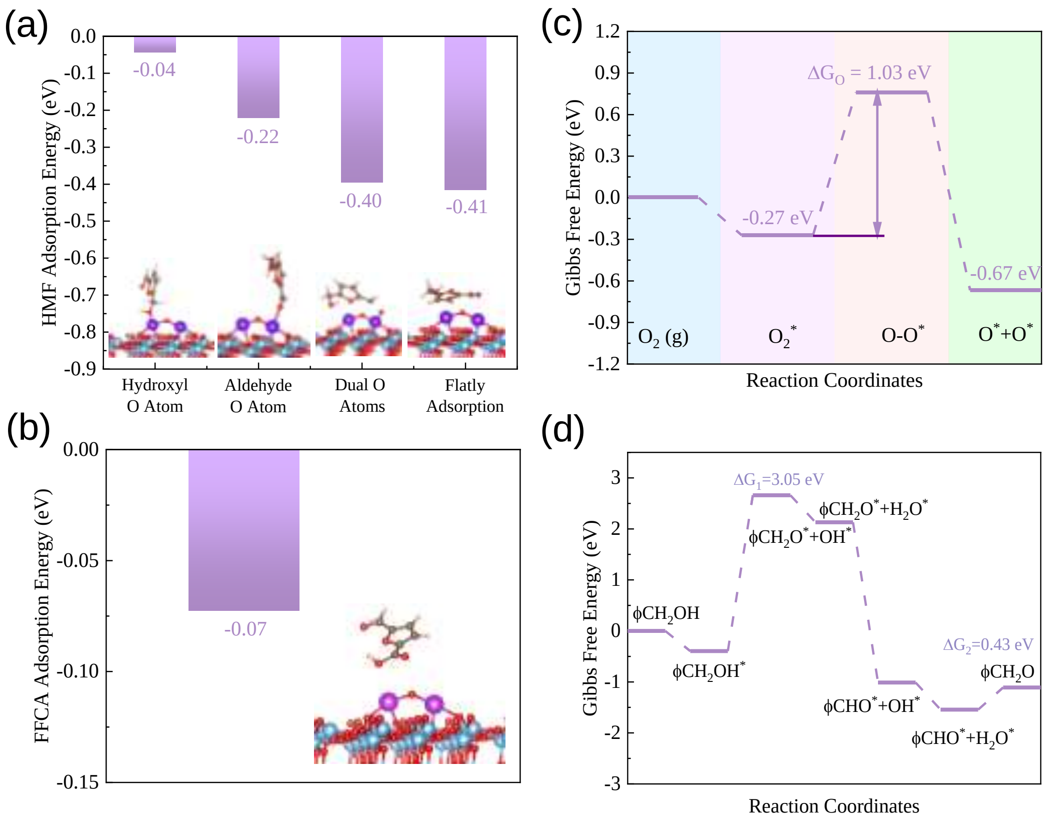

Figure S29 HMF (a) and FFCA (b) adsorption energies of different adsorption states on Bi/TiO_2; (c) Change of Gibbs free energy during O_2 dissociation, and (d) Change of Gibbs free energy during HMF initial oxidation to DFF over Bi/TiO_2.

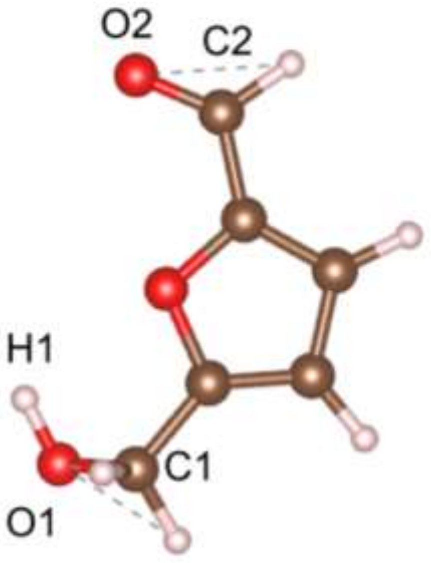

Figure S30 The structure and marked bonds of HMF.

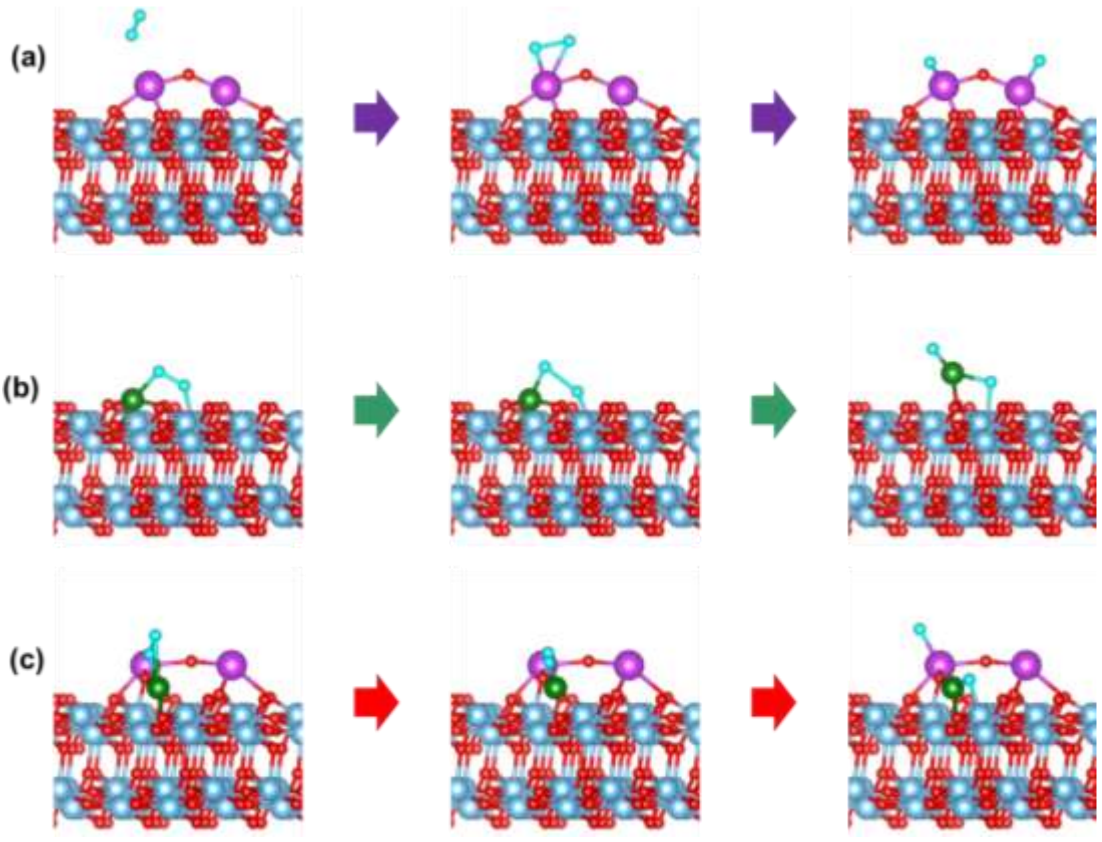

Figure S31 Model diagram of O_2 adsorption and dissociation (O* → O–O* → O* + O*) on (a) Bi/TiO_2, (b) Pt/TiO_2 and (c) $Pt\text{-}Bi/TiO_2$.

(1) $O_2 + 2* \leftrightarrow 2O*$

(2) $R - CH_2OH + * \leftrightarrow R - CH_2OH*$

(3) $R - CH_2OH * + O * \rightarrow R - CH_2O * + OH*$

(4) $R - CH_2O * + O * \rightarrow R - CHO * + OH*$

(5) $R - CHO * \rightarrow R - CHO + *$

(6) $OH * + OH * \rightarrow H_2O + O*$

(7) $H_2O * \leftrightarrow H_2O + *$

Scheme S1 Proposed primary element reaction pathways for aerobic oxidation of HMF to DFF.

SUPPLEMENTARY TABLES

Table S1 The binding energies and peak areas of the Ti 2p XPS spectra for TiO_2-based materials.

Catalyst	Ti(III)		Ti(IV)		Ti(III)/Ti(IV)
	$2p_{3/2}$	$2p_{1/2}$	$2p_{3/2}$	$2p_{1/2}$	
Pt/TiO_2	1.8% (457.0 eV)	0.5% (462.8 eV)	66.2% (458.5 eV)	31.5% (464.3 eV)	0.03
Bi/TiO_2	1.9% (457.0 eV)	0.6% (462.3 eV)	66.1% (458.5 eV)	31.4% (464.2 eV)	0.03
$Pt\text{-}Bi/TiO_2$	3.1% (457.0 eV)	1.5% (462.9 eV)	65.2% (458.6 eV)	30.2% (464.4 eV)	0.05

Table S2 The binding energies of the Pt 4f XPS spectra and the corresponding peak areas for TiO_2-based materials.

Catalyst	Pt(0)		Pt(Ⅱ)		Pt(Ⅳ)		$Pt^0/Pt^{\delta+}$
	$4f_{7/2}$	$4f_{5/2}$	$4f_{7/2}$	$4f_{5/2}$	$4f_{7/2}$	$4f_{5/2}$	
Pt/TiO_2	10.8% (70.6)	8.5% (74.4)	11.9% (72.0)	10.7% (75.5)	36.3% (74.2)	21.8% (77.0)	0.22
$Pt\text{-}Bi/TiO_2$	2.8% (70.2)	2.2% (73.6)	22.8% (71.0)	20.9% (75.3)	34.2% (74.1)	17.1% (76.9)	0.05

The value shown in parentheses is the binding energies (eV) of the Pt 4f.

Table S3 The binding energies and peak areas of the Bi 4f XPS spectra for TiO_2-based materials.

Catalyst	Bi(0)		Bi(Ⅲ)		Bi(0)/Bi(Ⅲ)
	$4f_{7/2}$	$4f_{5/2}$	$4f_{7/2}$	$4f_{5/2}$	
Bi/TiO_2	10.7% (159.3 eV)	8.5% (164.6 eV)	45.2% (158.9 eV)	35.6% (164.2 eV)	0.24
Pt-Bi/TiO_2	27.0% (156.9 eV)	21.6% (162.2 eV)	28.5% (157.4 eV)	22.8% (163.0 eV)	0.95

Table S4 Curve-fit parameters for Pt *L3*-edge EXAFS. (N coordination number, R bonding length)

Sample	Path	*d*/Å	*N*	*R*/Å	σ^2/Å^2	S_0^2
Pt Foil [a]	Pt-Pt	2.788 [b]	10.0	2.777	0.003	0.851
PtO_2 [c]	Pt-O	2.015 [d]	6.17	2.014	0.002	0.851 [e]
	Pt-Pt	3.157 [d]	8.46	3.111	0.005	0.851 [e]
	Pt-O	3.738 [g]	8.14	3.656	0.004	0.851 [e]
Pt/TiO_2 [f]	Pt-O	2.012 [g]	2	1.973	0.004	0.851 [e]
	Pt-Ti	2.742 [h]	1	2.626	0.022	0.851 [e]
	Pt-O	3.478 [g]	4	3.444	0.009	0.851 [e]
Pt-Bi/TiO_2 [i]	Pt-O	2.012 [g]	1	1.982	0.003	0.851 [e]
	Pt-Bi	2.767 [j]	1	2.752	0.001	0.851 [e]
	Pt-O	3.478 [g]	4	3.501	0.035	0.851 [e]

[a] ΔE for Pt Foil was refined as a global fit parameter, returning a value of 8.79 eV. Data ranges: $2.5 \leq k \leq 12.5$ Å^{-1}, $2 \leq R \leq 3$ Å. The number of variable parameters is 4 out of a total of 6.02 independent data points.

[b] The distances of Pt-Pt are from the crystal structure (*F*m-3m, *a*=*b*=*c*=3.94, *α*=*β*=*γ*=90).

[c] ΔE for PtO_2 was refined as a global fit parameter, returning a value of 9.77 eV. Data ranges: $3 \leq k \leq 12$ Å^{-1}, $1 \leq R \leq 4$ Å. The number of variable parameters is 10, out of a total of 16.86 independent data points.

[d] The distances of Pt-O and Pt-Pt are from the crystal structure (*Pnnm*, *a*=4.5101, *b*=4.5472, *c*=3.1568, *α*=*β*=*γ*=90).

[e] The Debye-Waller factor was fixed as 0.851, according to the Pt Foil fitting results.

[f] ΔE for Pt/TiO_2 was refined as a global fit parameter, returning a value of 8.08 eV. Data ranges: $2.5 \leq k \leq 11$ Å^{-1}, $1 \leq R \leq 3.5$ Å. The number of variable parameters is 7 out of a total of 13.37 independent data points.

[g] The distances of Pt-O are from the crystal structure (*P*42/mnm, *a*=*b*=4.5916; *c*=3.25, *α*=*β*=*γ*=90).

[h] The distances of Pt-Ti are from the crystal structure (P1, *a*=2.7682, *b*=4.6003, *c*=4.8503, *α*=*β*=*γ*=90). The coordination numbers were fixed as 2, 1, 4 for corresponding paths.

[i] ΔE for Pt-Bi/TiO_2 was refined as a global fit parameter, returning a value of 9.22 eV. Data ranges: $2.5 \leq k \leq 11$ Å^{-1}, $1 \leq R \leq 3$ Å. The number of variable parameters is 8 out of a total of 10.56 independent data points.

[j] The distances of Pt-Bi are from the crystal structure (P1, a=4.3823, b=4.3823, c=5.5342, *α*=*β*=*90*, *γ*=120). The coordination numbers were fixed as 1, 1, 4 for corresponding paths.

Table S5 The average Bader charge value and average valence state of Pt and Bi on different catalyst modes as shown in **Figure S24**.

Sample	Atom	Bader charge	State value
Pt/TiO_2	O1	6.938	
	O2	6.934	
	Pt	9.235	1.530
Bi/TiO_2	O1	7.053	
	O2	7.016	
	O3	7.039	
	Bi1	3.800	2.400
	Bi2	3.914	2.172
Pt-Bi/TiO_2	O1	7.021	
	O2	7.058	
	O3	6.981	
	O4	6.964	
	O5	7.060	
	O6	7.041	
	Pt	10.081	-0.163
	Bi1	3.631	2.738
	Bi2	3.316	3.368

Table S6 The structural parameters of bonds of HMF absorbed on Pt/TiO_2 and Pt-Bi/TiO_2 in different forms.

Location		H1-O1 (Å)	C1-O1 (Å)	C2-O2 (Å)	H1-O1-C1 (°)
HMF model Without adsorption		0.9753	1.4305	1.2289	107.8706
Pt/TiO_2	Hydroxyl adsorption	0.9765	1.4387	1.2305	109.2894
	Aldehyde adsorption	0.9752	1.4341	1.2310	108.1354
	Dual adsorption	0.9776	1.4384	1.2257	109.5330
	Flatly adsorption	0.9746	1.4316	1.2135	109.1388
Bi/TiO_2	Hydroxyl adsorption	0.9764	1.4381	1.2286	109.2826
	Aldehyde adsorption	0.9727	1.4381	1.2299	108.1123
	Dual adsorption	0.9759	1.4364	1.2233	109.4710
	Flatly adsorption	0.9742	1.4292	1.2119	108.8756
Pt-Bi/TiO_2	Hydroxyl adsorption	0.9836	1.4456	1.2301	106.0368
	Aldehyde adsorption	0.9746	1.4332	1.2276	108.0476
	Dual adsorption	0.9850	1.4461	1.2479	108.4165
	Flatly adsorption	0.9765	1.4190	1.2300	107.7242

REFRENCE

(1) Xu H, Wang Z, Miao Z *et al.* Correlation between acidity and catalytic performance of mesoporous zirconium oxophosphate in phenylglyoxal conversion. *ACS Sustain Chem Eng* 2019; **7**: 8931-42.

(2) Ravel B, Newville M. ATHENA, ARTEMIS, HEPHAESTUS: data analysis for X-ray absorption spectroscopy using IFEFFIT. *J Synchrotron Radiat,* 2005*;* **12**: 537-41.

(3) Feng C, Gao Q, Xiong G *et al.* Defect engineering technique for the fabrication of $LaCoO_3$ perovskite catalyst via urea treatment for total oxidation of propane. *Appl Catal B Environ* 2022; **304**: 121005.

(4) Mao X, Gu Z, Yan C *et al.* Unlocking the potential of ruthenium catalysts for nitrogen fixation with subsurface oxygen. *J Mater Chem A* 2021; **9**: 6575-82.

(5) Liao X, Lu R, Xia L *et al.* Density functional theory for electrocatalysis. *Energy Environ Mater* 2022; **5**: 157-85.

(6) Kong W, Xu J, Tong Y *et al.* Construction of dual active sites for efficient alkaline hydrogen evolution: single-metal-atoms supported on BC_2N monolayers. *Phys Chem Chem Phys* 2022; **24**: 29141-50.

(7) Wu ZY, Karamad M, Yong X *et al.* Electrochemical ammonia synthesis via nitrate reduction on Fe single atom catalyst. *Nat Commun* 2021; **12**: 2870.
