# Supplementary material for: Strong metal-support interaction induced by Pt-O-Bi bonding in mesoporous anatase TiO2 for base-free catalytic biomass valorization
Source: Natl Sci Rev. 2025 Aug 26;12(10):nwaf327. doi: 10.1093/nsr/nwaf327 (PMC12491999; doi:10.1093/nsr/nwaf327)
Supplement: nwaf327_Supplemental_Files [file nwaf327_supplemental_files.zip › Teaser Text.docx]

This work develops a Pt single-atom dominant Pt–Bi/TiO_2_ catalyst with high activity for base-free oxidation of biomass-derived HMF, and elucidates the complex metal–support interactions involved.
